# Construction of Two Stable Co(II)-Based Hydrogen-Bonded Organic Frameworks as a Luminescent Probe for Recognition of Fe^3+^ and Cr_2_O_7_^2−^ in H_2_O

**DOI:** 10.3390/molecules26195955

**Published:** 2021-09-30

**Authors:** Qi-Ying Weng, Ya-Li Zhao, Jia-Ming Li, Miao Ouyang

**Affiliations:** 1 Guangxi Key Laboratory of Green Chemical Materials and Safety Technology, College of Petroleum and Chemical Engineering, Beibu Gulf University, Qinzhou 535011, China; qy13977735754@163.com (Q.-Y.W.); ylzhao@bbgu.edu.cn (Y.-L.Z.); 2 College of International Studies, Beibu Gulf University, Qinzhou 535011, China; 3 School of Chemical and Environmental Engineering, Hanshan Normal University, Chaozhou 521041, China

**Keywords:** hydrogen-bonded organic framework (HOFs), Co(II)-based complex, synthesis, photoluminescence, crystal structure

## Abstract

A pair of cobalt(II)-based hydrogen-bonded organic frameworks (HOFs), [Co(pca)_2_(bmimb)]*_n_* (**1**) and [Co_2_(pca)_4_(bimb)_2_] (**2**), where Hpca = *p*-chlorobenzoic acid, bmimb = 1,3-bis((2-methylimidazol-1-yl)methyl)benzene, and bimb = 1,4-bis(imidazol-1-ylmethyl)benzene were hydrothermally synthesized and characterized through infrared spectroscopy (IR), elemental and thermal analysis (EA), power X-ray diffraction (PXRD), and single-crystal X-ray diffraction (SCXRD) analyses. X-ray diffraction structural analysis revealed that **1** has a one-dimensional (1D) infinite chain network through the deprotonated pca^−^ monodentate chelation and with a μ_2_-bmimb bridge Co(II) atom, and **2** is a binuclear Co(II) complex construction with a pair of symmetry-related pca^−^ and bimb ligands. For both **1** and **2**, each cobalt atom has four coordinated twisted tetrahedral configurations with a N_2_O_2_ donor set. Then, **1** and **2** are further extended into three-dimensional (3D) or two-dimensional (2D) hydrogen-bonded organic frameworks through C–H···Cl interactions. Topologically, HOFs **1** and **2** can be simplified as a 4-connected ***qtz*** topology with a Schläfli symbol {6^4^·8^2^} and a 4-connected ***sql*** topology with a Schläfli symbol {4^4^·6^2^}, respectively. The fluorescent sensing application of **1** was investigated; **1** exhibits high sensitivity recognition for Fe^3+^ (*K*_sv_: 10970 M^−1^ and detection limit: 19 μM) and Cr_2_O_7_^2−^ (*K*_sv_: 12960 M^−1^ and detection limit: 20 μM). This work provides a feasible detection platform of HOFs for highly sensitive discrimination of Fe^3+^ and Cr_2_O_7_^2−^ in aqueous media.

## 1. Introduction

Recently, a new class of materials named hydrogen-bonded organic frameworks (HOFs) has emerged as an exciting class of compounds stabilized by non-covalent hydrogen-bonding and π−π stacking interactions [1,2,3,4,5]. HOFs have attracted a significant deal of attention especially because of their broad applications, ranging from chemical sensing [6] and bioimaging [7,8] to lighting [9] and 3D optical storage [10,11]. The metal-organic frameworks (MOFs) and covalent-organic frameworks (COFs) are usually connected by coordination bonds or covalent bonds between atoms and show relatively high stabilities [12,13,14,15,16,17,18,19,20], while the HOFs are linked by weak non-covalent interactions, such as hydrogen bonds and π−π stacking interactions, and exhibit relatively low stabilities [21,22,23,24,25]. For HOFs, the solvent guests play important roles in the construction of the supermolecular network system. Once the solvent guests are removed, the supermolecular system is usually broken, and the HOFs collapse. Therefore, it is unsurprising that, although thousands of HOFs have been reported in the literature during the last two decades, examples of HOFs with permanent stable are very rare [26,27].

Hydrogen bonding, particularly within the coordination sphere, is another strategy which may lead to enhancement in the chemical stability at the nodes and therefore, of the framework materials themselves [28,29]. These interactions may act as support and rigidity of the coordination geometry of mononuclear coordination spheres, as well as provide extra thermodynamic and kinetic stabilization to meet the challenge of hydrolytic stability in these materials. However, a small subset of known MOF ligands contains suitable functionality for coordination sphere hydrogen bonding, which can provide new opportunities in ligand design [30,31,32,33,34].

To date, the halogen-containing carboxylic acid ligands, such as *p*-chlorobenzoic acid (Hpca), have afforded great attention in the preparation of HOFs for their outstanding features of versatile coordination fashions as well as potential hydrogen-bonding donors (C and O) and acceptors (Cl) [35,36,37,38,39,40,41,42,43]. A mononuclear complex can be afforded with the metal ions reaction with Hpca under appropriate conditions. Accordingly, C–H···Cl, C–Cl···π, C–H···π and π···π are excellent candidates for assembling mononuclear complex to HOFs in different ways, ranging from one-dimensional (1D) chains and two-dimensional (2D) sheets to three-dimensional (3D) porous structures [44,45,46,47,48,49,50,51,52]. Of further interest, *p*-chlorobenzoic acid featuring a chlorine in the 4-positions of the phenyl ring, as a derivative of benzoic acid, remains largely unexplored hitherto in the field of HOFs, compared with the well-studied ligands, benzoic acid.

**Scheme 1:**
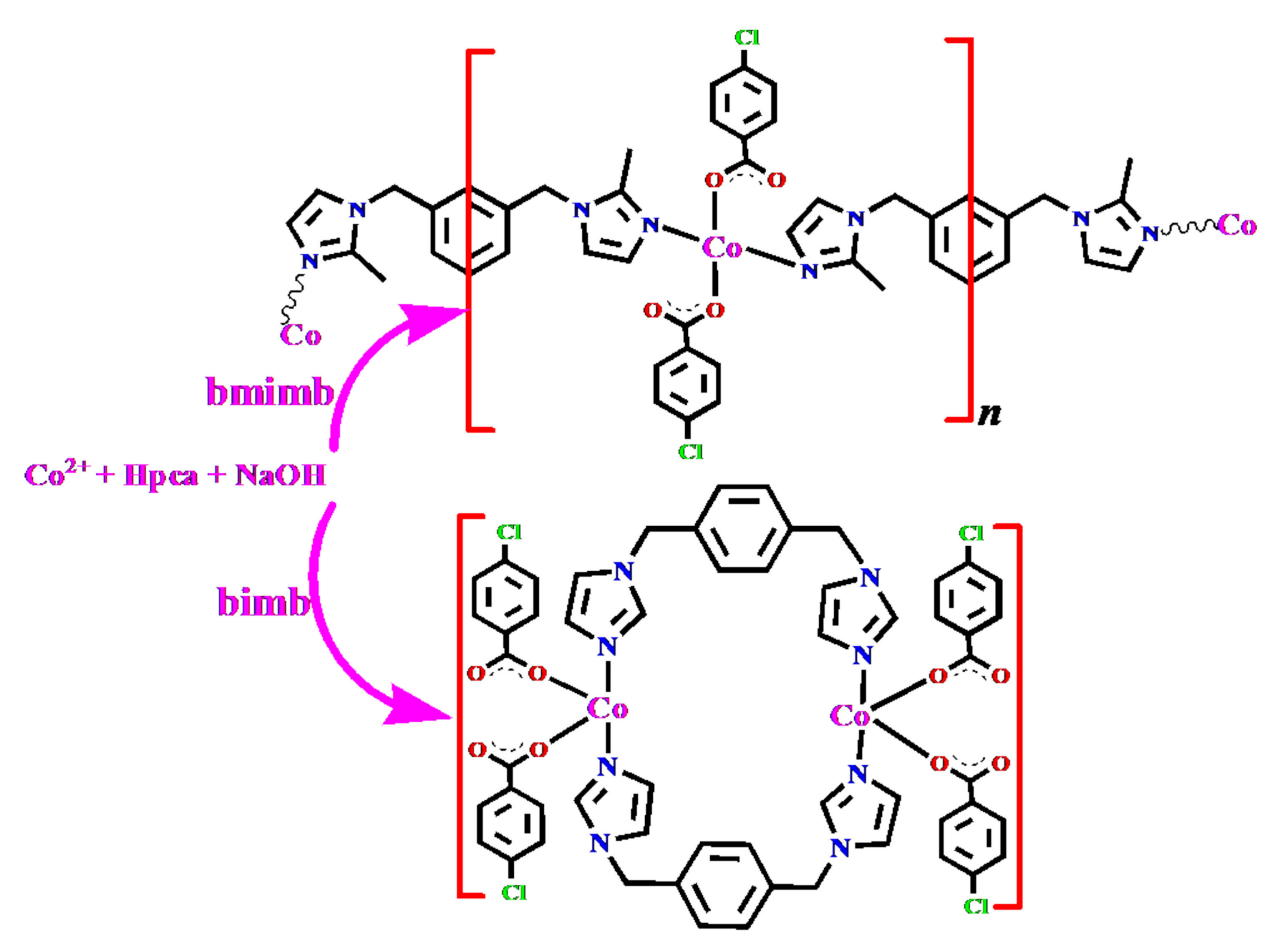
The route for synthesis of **1** and **2**.

Based on the above considerations and ongoing our research work in this area [53,54,55,56,57,58,59], we utilized Hpca organic ligands to act as the building blocks. Two Co(II)-based HOFs, [Co(pca)_2_(bmimb)]*_n_* (**1**) and [Co_2_(pca)_4_(bimb)_2_] (**2**) were successfully synthesized by hydrothermal methods and characterized by infrared spectrum (IR) (Figure S1), elemental analysis (EA), power X-ray diffraction (PXRD), single-crystal X-ray diffraction (SCXRD) and thermogravimetry (TG) (Scheme 1). In addition, the solid-state luminescence properties of **1** and **2**, and the fluorescent detection of cations/anions of **1,** were investigated at room temperature in detail.

## 2. Results and Discussion

### 2.1. Description of the Structure of [Co(pca)_2_(bmimb)]_n_ (***1***)

**Figure 1:**
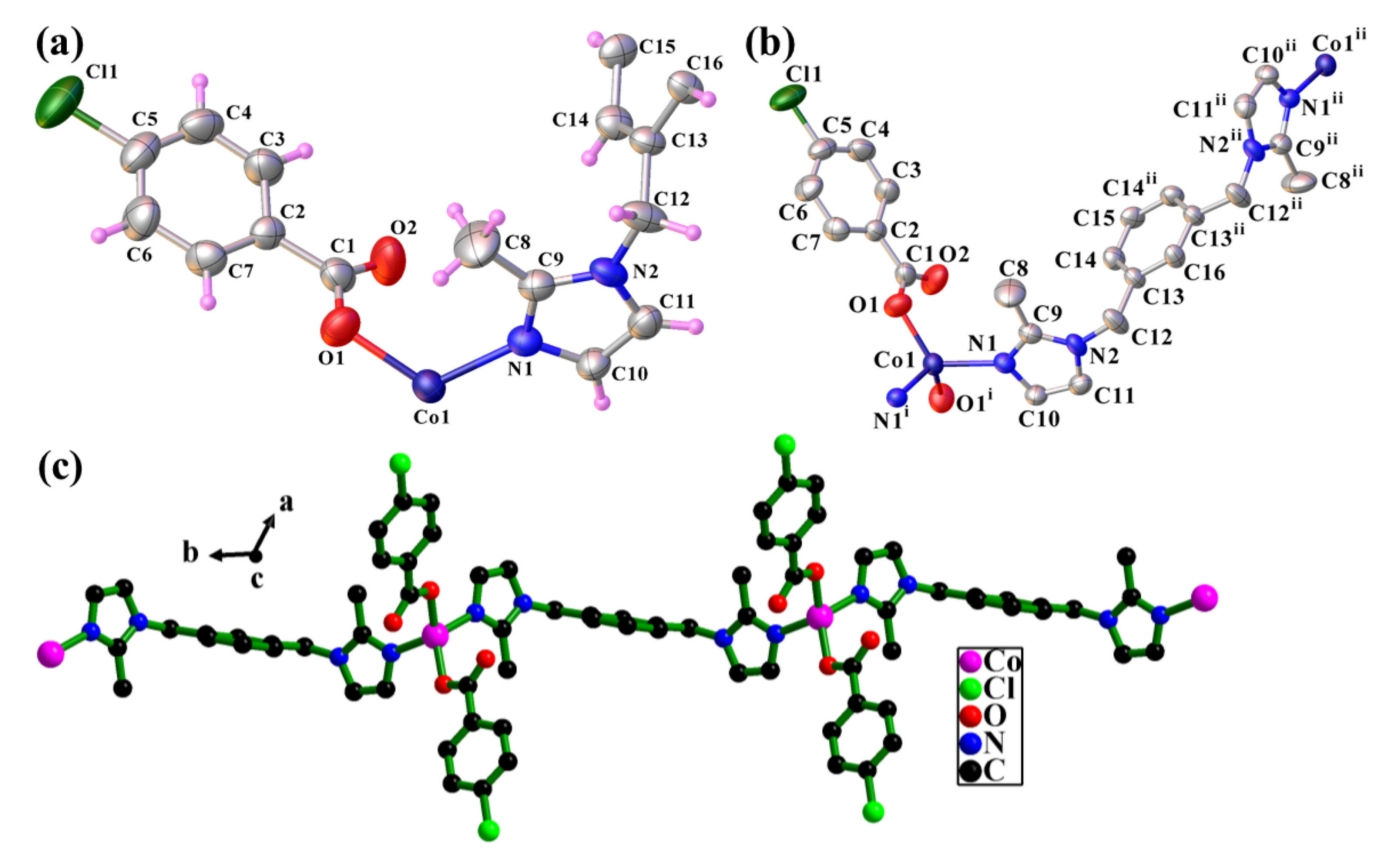
(**a**) The asymmetric unit of **1**. (**b**) The coordination environment for Co^2+^ in **1**. (**c**) The 1D chain framework in **1**.

X-ray single crystal diffraction revealed that **1** is a one-dimensional coordination polymer and crystallizes in the monoclinic system with space group *P*2_1_/*n* (Table S1). The asymmetric unit of **1** is comprised of one Co^2+^, one deprotonated monodentate chelating ligand pca^−^ and half of a neutral auxiliary ligand, bmimb (Figure 1a). Each Co ion is tetra-coordinated with two O atoms (O1 and O1^i^, symmetry code i seen in Table S2) and two N atoms (N1 and N1^i^) from two symmetry-related pac^−^ and bmimb ligands, resulting in a slightly distorted tetrahedron geometry (Figure 1b). The Co–O bond distance is 1.948(4) Å, and the Co–N distance is 2.048(4) Å (Table S2). 

The detailed coordination mode of pca^−^ and bmimb ligands is shown in Figure 1c. Notably, the carboxyl group in pca^−^ adopts the mondenate type of coordination mode η^1^:η^0^, while the bmimb ligand adopts a normal μ_2_ bridge coordination fashion. Two symmetry-related pca^−^ ligands bond to each Co^2+^ cation by the upside-down fashion through their deprotonated carboxylate O-atom, resulting in substructural blocks {[Co(pca^−^)]_2_}^2+^. These neighboring {[Co(pca^−^)]_2_}^2+^ are further interconnected through the μ_2_-bmimb bridge to form a one-dimensional infinite framework with a Co···Co separation of 14.02(4) Å.

**Figure 2:**
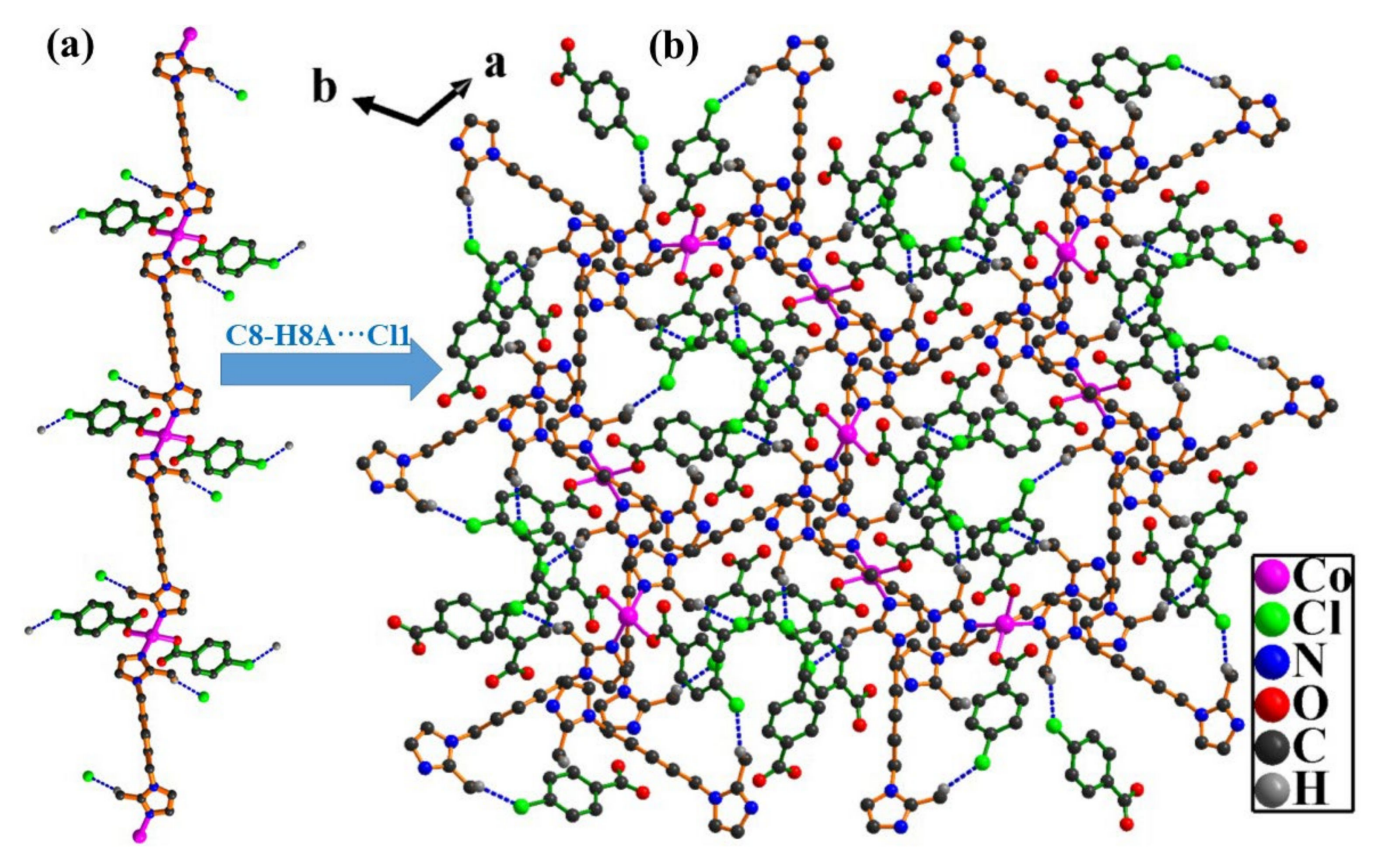
(**a**) The enlarged C8–H8A···Cl1 interactions in the 1D chain framework for **1**. (**b**) View of the 3D Co-based hydrogen-bonded framework assembled with C–H···Cl interactions in **1** from *c* direction.

There are hydrogen-bonding interactions (calculated by PLATON software) [60] that stabilize its structure and result in the formation of a three-dimensional network (Figure 2 and Table S3). In particular, interchain hydrogen bonds C–H···Cl stabilized the crystal structure of **1**; these were formed between the methyl group of bmimb ligand (-CH_3_, C8), which is the donor, and the chlorine of the pca^−^ (Cl1), which acts as the acceptor (C8···Cl1^viii^ = 3.676(7), H8A···Cl1^viii^ = 2.84, C8-H8A···Cl1^viii^ = 147°, symmetry code viii −*x*+*y*+1, −*x*+2, *z*−1/3). The bond distance between H8A and Cl1 is 2.840 Å; the bond angle of C8–H8A···Cl1 is 147°, which exceeds the preferred minimum hydrogen bond angle of 110°; and the distance between C8 and Cl1 is 3.676(7) Å, which is slightly greater than the sum of the van der Waals radii of C and Cl (3.52 Å) [61]. Therefore, it belongs to a strong C–H···Cl hydrogen bond, compared with the reported literature [62,63,64,65,66,67,68].

**Figure 3:**
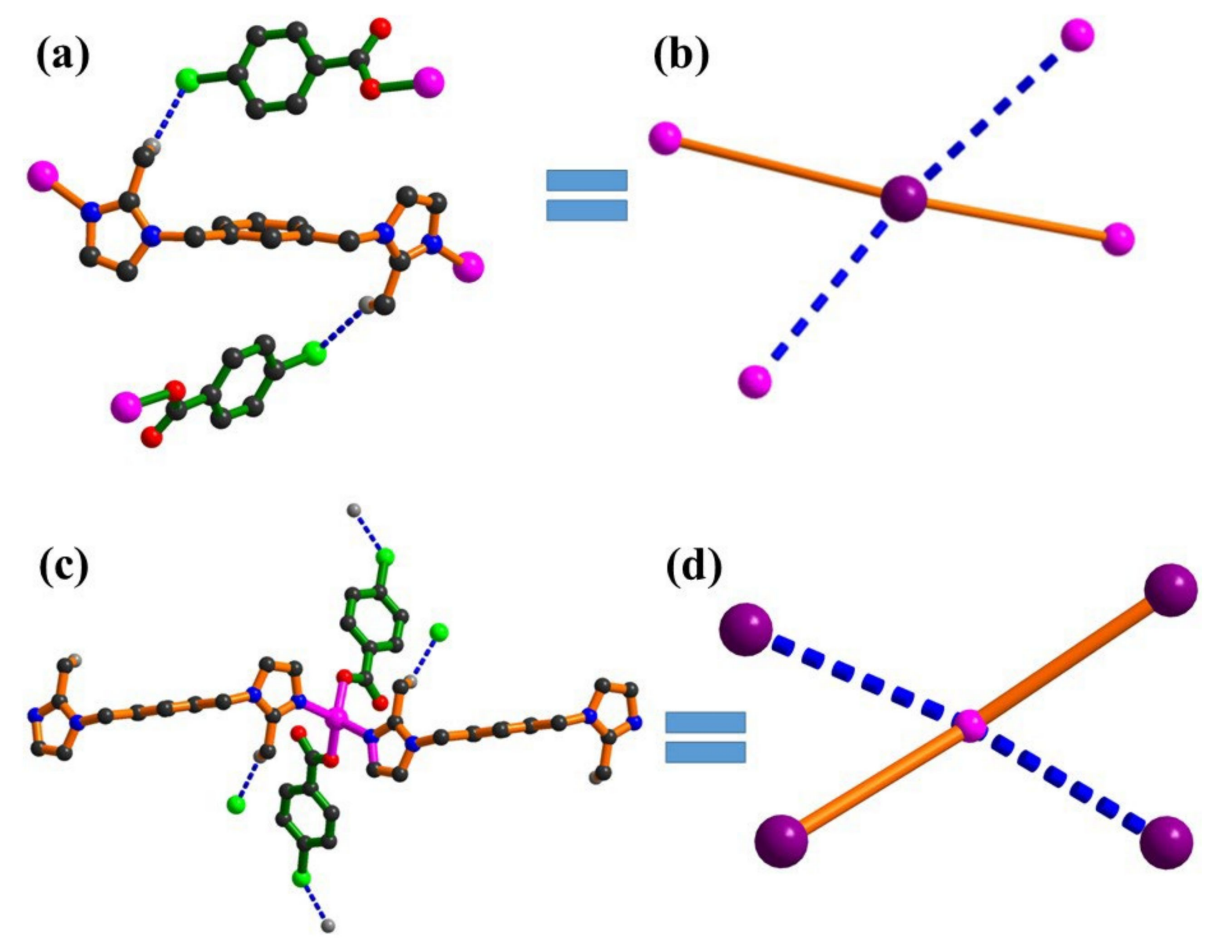
(**a**) The enlarged C8–H8A···Cl1 interactions in **1**. (**b**) The [Co_4_(bmimb)(pca)_2_]^6+^ unit simplified as a four connected node (violet ball represents the core of the bmimb ligand, and pink ball represents Co^2+^; solid lines represent bmimb ligands, and dash lines represent C–H···Cl hydrogen bonds). (**c**) The Co(pca)_2_(bmimb)_2_ unit linked by four symmetry-related C8–H8A···Cl1 interactions in **1**. (**d**) The Co(pca)_2_(bmimb)_2_ unit simplified as a four connected node (violet ball represents the core of the bmimb ligand, and pink ball represents Co^2+^; solid lines represent bmimb ligands, and dash lines represent C–H···Cl hydrogen bonds).

**Figure 4:**
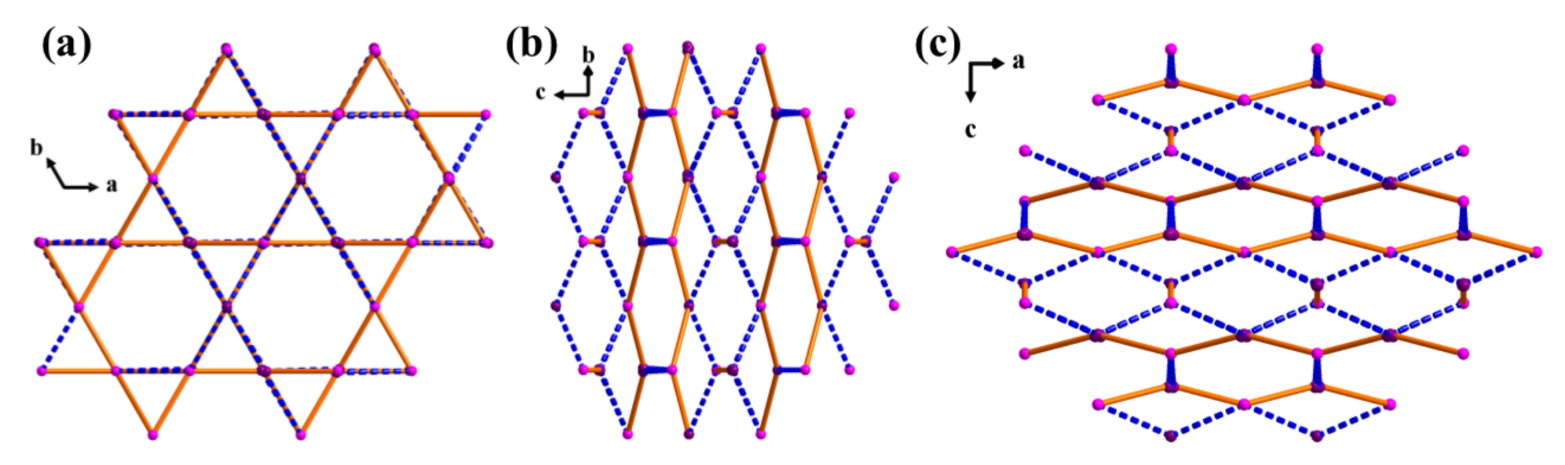
(**a**–**c**): View of the simplified 3D Co-based hydrogen-bonded framework of **1** from *c*, *a* and *b* directions (violet ball represents the core of bmimb ligand, and pink ball represents Co^2+^; solid lines represent bmimb ligands, and dash lines represent C–H···Cl hydrogen bonds).

Each bmimb links two pca^−^ ligands with two inverse centrosymmetric groups -CH_3_ as a hydrogen-bonded substructural unit ([Co_4_(pca)_2_(bmimb)]^6+^) (Figure 3a), which is further extended by bmimb ligands, resulting in a 3D Co-based hydrogen-bonded organic frameworks. From the topological point of view, each [Co_4_(pca)_2_(bmimb)]^6+^ can be considered to act as a 4-connected node, and each {Co^2+^} links 2 pca^−^ and 2 bmimb molecules, which also can be considered a 4-connected node in **1** (Figure 3). Interestingly, the 3D Co-based hydrogen-bonded organic frameworks can be topologically simplified as a 4-connected 2-nodal ***qtz*** net with a Schläfli symbol {6^4^·8^2^} (Figure 4) as analyzed with the TOPOS 4.0 program [69].

Furthermore, between adjacent 1D chains, there exists a C–Cl···π stacking interaction between the symmetry-related pca^−^ (C5–Cl5 and C2–C3–C4–C5–C6–C7, Cg2^ix^, symmetry code: ix 2−*y*, 1+*x*−*y*, 1/3+*z*). The angle C5–Cl5···Cg2^ix^ is 153.1(3)°, with Cl5···Cg2^ix^ and C5···Cg2^ix^ distances of 3.843(4) Å and 5.454(7) Å, respectively. Thus, these hydrogen bonds insert and inter-lock the 1D chain in reversely alternating parallel arrangements, defining 3D HOFs supporting the supramolecular architecture through C–H···O, C–H···N, C–H···Cl and C–Cl···π interactions. The unit cell contains no residual solvent accessible void calculated by PLATON program [60].

### 2.2. Description of the Structure of [Co_2_(pca)_4_(bimb)_2_] (***2***)

**Figure 5:**
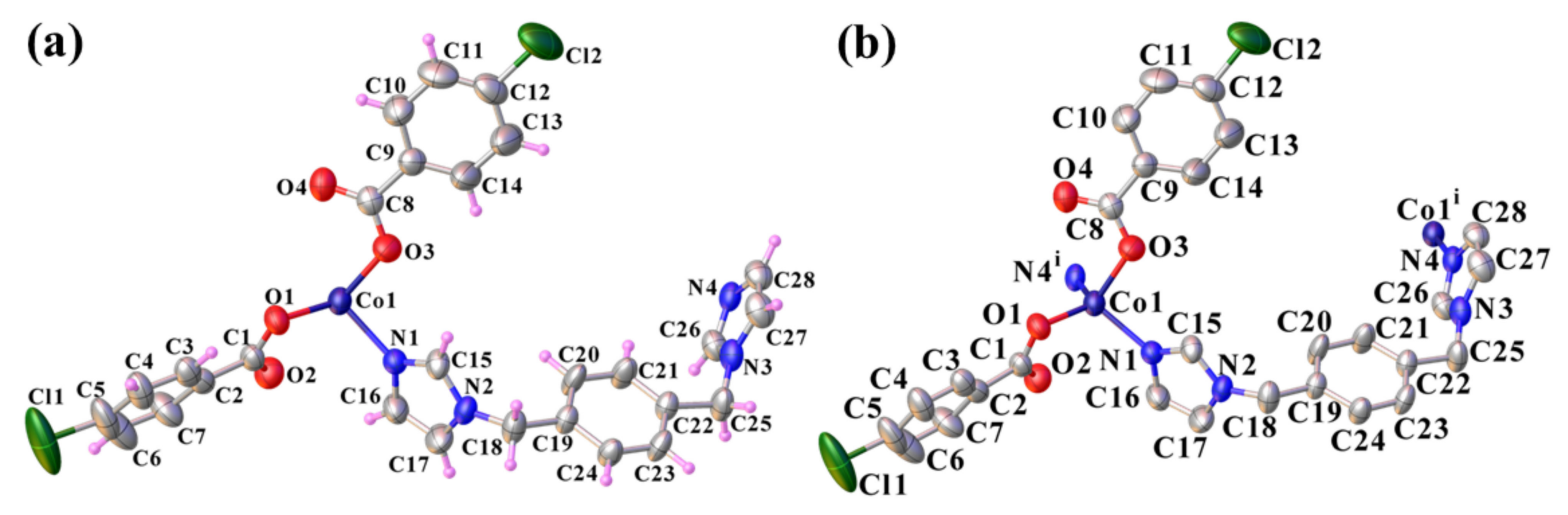
(**a**) The asymmetric unit of **2**. (**b**) The coordination environment for Co^2+^ in **2**.

**Figure 6:**
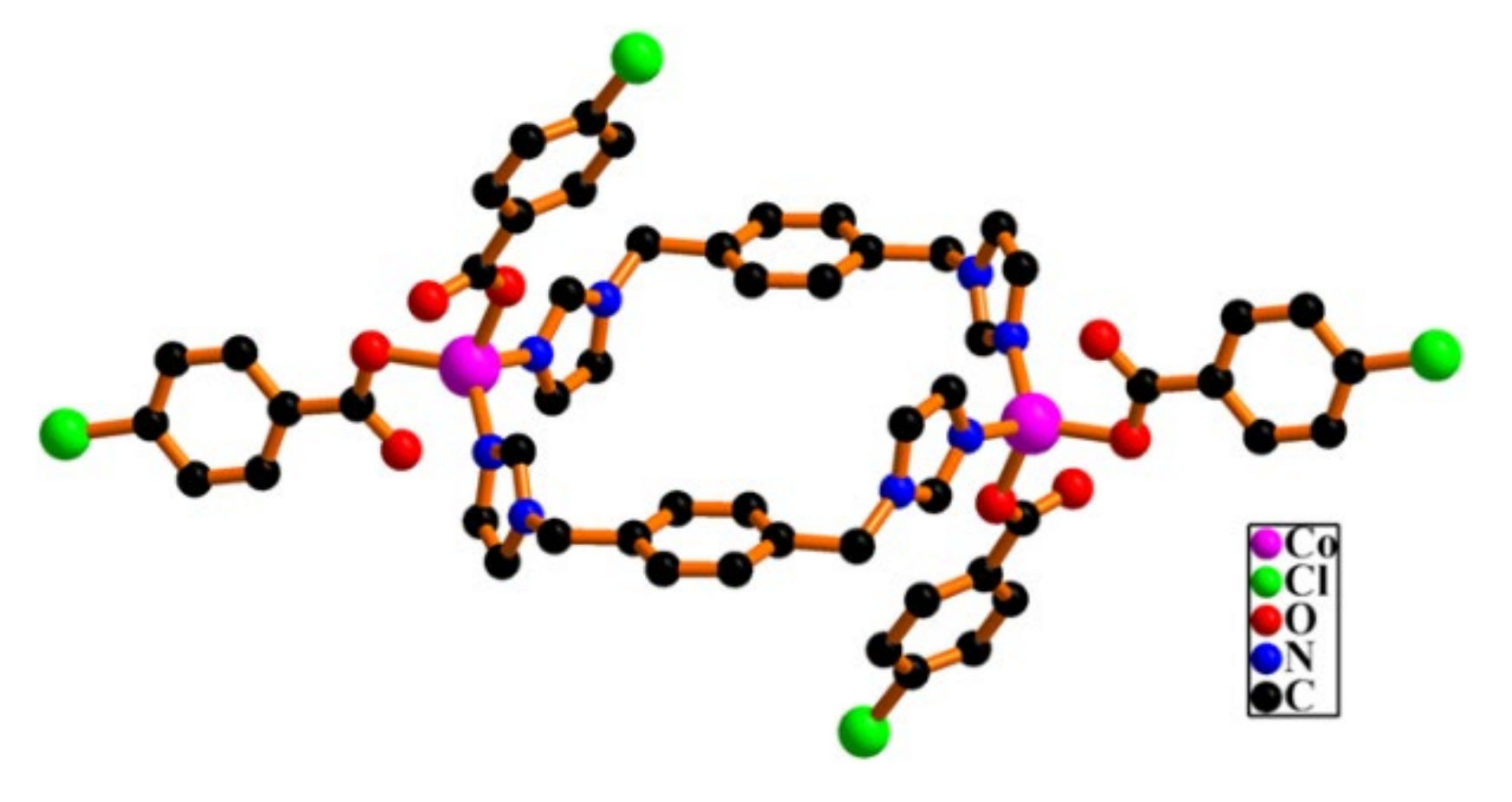
The [Co_2_(pca)_4_(bimb)_2_] structure unit.

Definitely different from **1**, X-ray single crystal diffraction indicates that **2** is a centrosymmetric binuclear cobalt complex and crystallizes in the triclinic system with space group *P*-1 (Table S1). The asymmetric unit of **2** consists of a half-molecule of the formula [Co_2_(pca)_4_(bimb)_2_] with one Co^2+^, one bimb, and two discrete pca^−^ ligands (Figure 5a). The coordination environment of each Co ion (shown in Figure 5b and Figure 6) contains two O atoms (O1 and O3) from two different pac^−^ ligands, and two N-atom donors (N1 and N4^i^, symmetry code i: 1−*x*, −*y*, 1−*z*) from two symmetry-related μ_2_-bridging bimb, resulting in a slightly distorted tetrahedron geometry. In comparison, **2** has two independent pca^−^ ligands, while **1** has one, and **2** has one bimb, while **1** has a half of symmetry-related bridging ligand. It is these different composition and coordination modes that lead to the formation of a completely different structure from compound **1**. The Co–O bond distances are 1.961(3) and 1.978(3) Å, and the Co–N distances are 2.013(4) and 2.028(5) Å (Table S2). The two halves of each independent dimer are related by a crystallographic inversion center, which lies at the center of the ring formed by the two Co atoms and the two bimb ligands. The Co1···Co1^i^ separation is 10.85(7) Å, distance that is too long for a metal–metal bond. 

**Figure 7:**
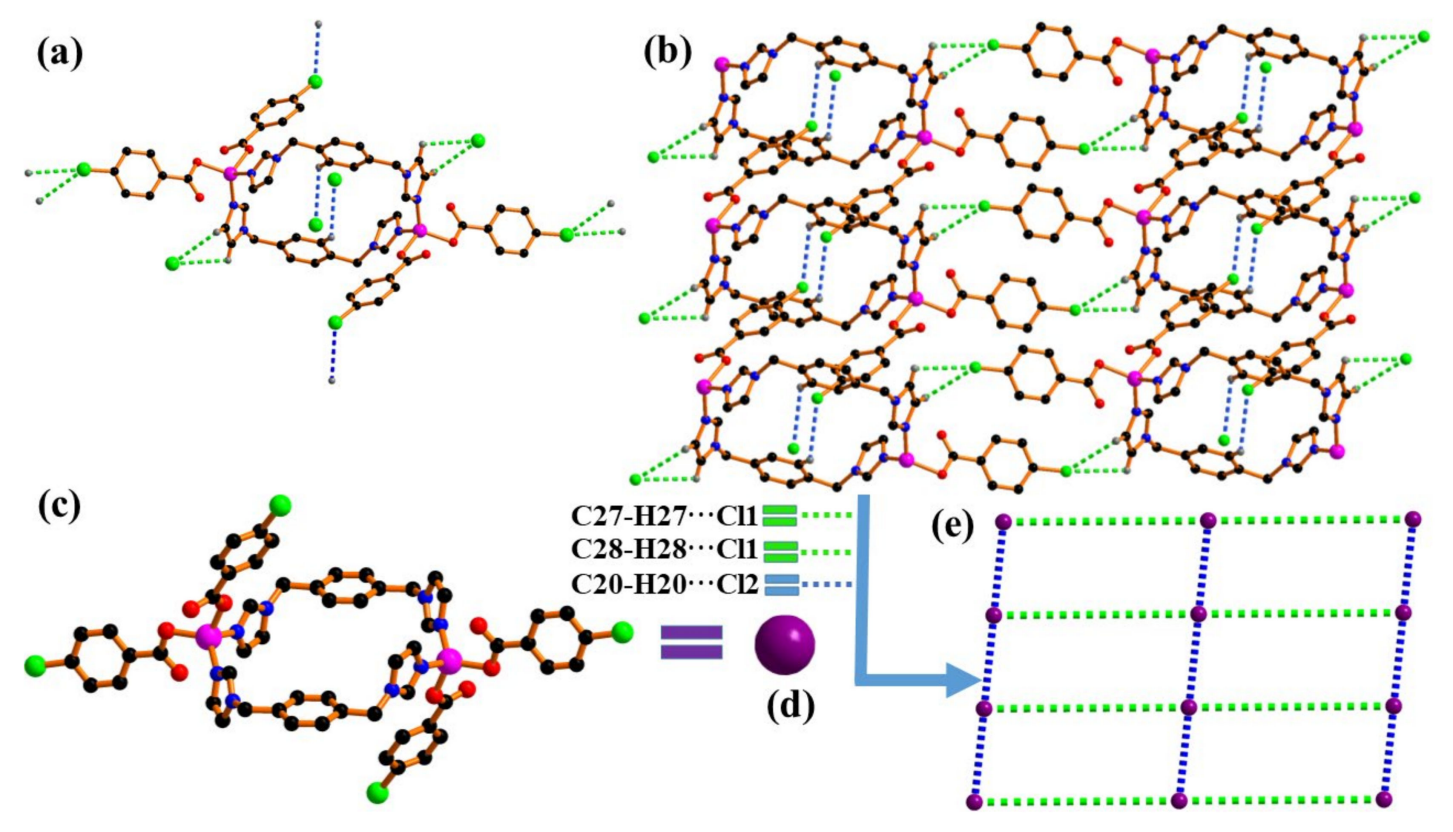
(**a**) The C–H···Cl interactions in **2** (bright green dash lines represent C20–H20···Cl2 hydrogen bonds, and blue dash lines represent C27–H27···Cl1 and C28–H28···Cl1 hydrogen bonds). (**b**) The 2D hydrogen-bonded organic framework constructed by C–H···Cl interactions in **2**. (**c**) The binuclear cobalt structure of **2**. (**d**) The C–H···Cl interactions simplified as a linker, and the [Co_2_(pca)_4_(bimb)_2_] simplified as a node. (**e**) The simplified Co-based HOFs in **2**.

**Figure 8:**
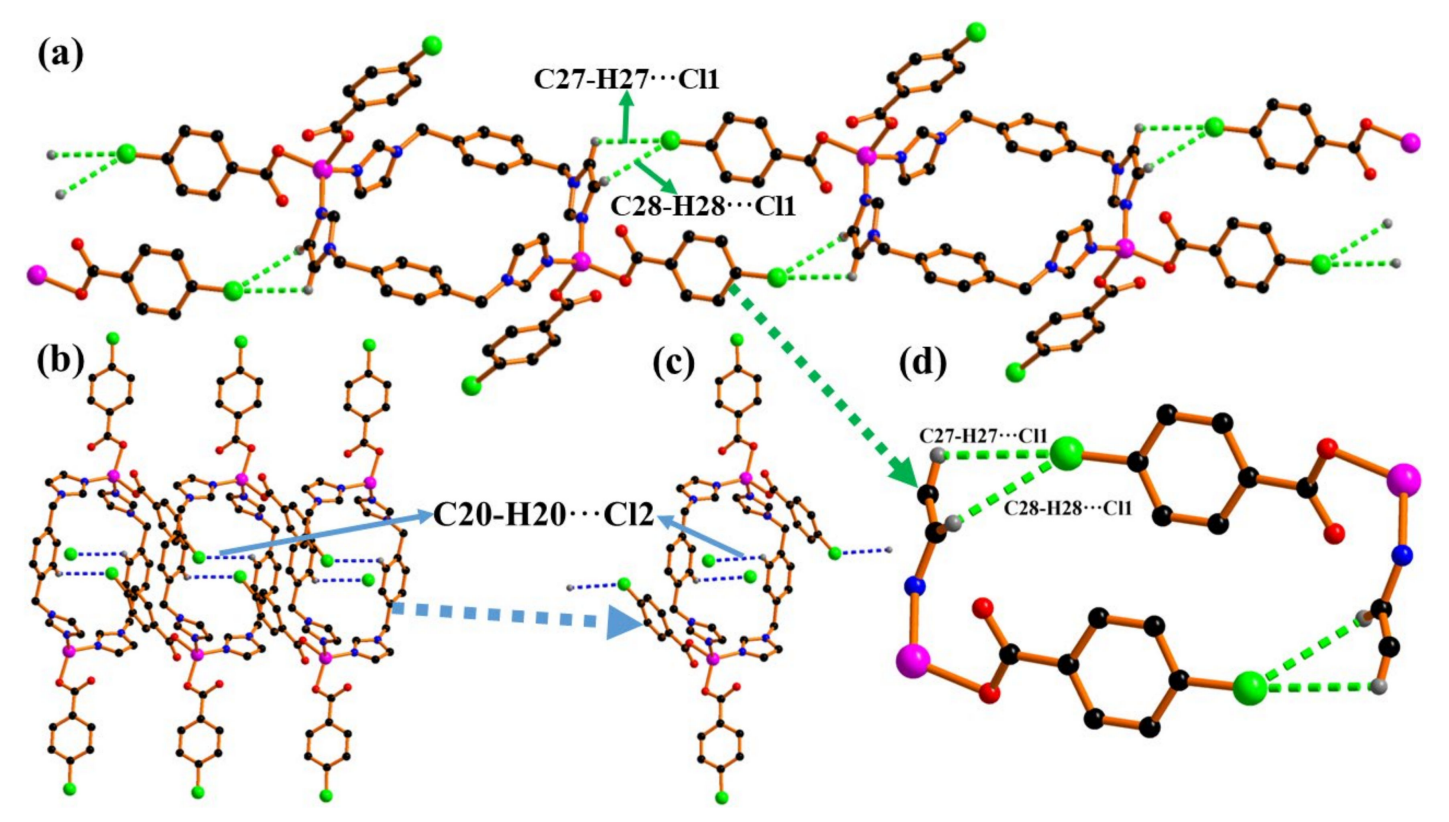
(**a**) The 1D chain constructed by C27–H27/C28–H28···Cl1 interactions in **2**. (**b**) The 1D chain constructed by a pair of C20–H20···Cl2 interactions in **2**. (**c**) The enlarged C20–H20···Cl2 interactions in **2**. (**d**) The enlarged C27–H27/C28–H28···Cl1 interactions in **2**.

In the crystal, intermolecular C–H···Cl hydrogen bonds connected the pca^−^ and the bimb ligands together with the phenyl and imidazole carbon atoms of bimb (C20, C27 and C28, donor), and the pca^−^ terminal chlorine atoms (Cl1 and Cl2, acceptor) (Figure 7 and Table S3). Interestingly, the C27–H27···Cl1 and C28–H28···Cl1 staggered the [Co_2_(pca)_4_(bimb)_2_] along the *a*-axis direction, while the C20–H20···Cl2 extended the [Co_2_(pca)_4_(bimb)_2_] along the *b*-axis, resulting in two mutually perpendicular 1D hydrogen-bonded chains (Figure 8a,b). 

The bond distances between H27/H28 and Cl1 are 3.175 Å and 3.125 Å; the bond angles of C27–H27/C28–H28···Cl1 are 104° and 107°, which fall slightly below the preferred minimum hydrogen bond angle of 110°; and the distances between C27/C28 and Cl1 are 3.522(6) Å and 3.511(6) Å, which are almost the same as the sum of the van der Waals radii of C and Cl (3.52 Å) [61]. The bond distance between H20 and Cl2 is 3.308 Å; the bond angle of C20–H20···Cl2 is 122°, which falls the preferred minimum hydrogen bond angle of 110°; and the distance between C20 and Cl2 is 3.884(6) Å, which is greater than the sum of the van der Waals radii of C and Cl (3.52 Å). Therefore, it is a very weak C–H···Cl hydrogen bond, according to the documented data [62,63,64,65,66,67,68].

In the asymmetric unit, each bimb adopts benzene (C20–H20···Cl2) and one of the two unsymmetrical groups imidazole (C27–H27···Cl1, C28–H28···Cl1) involved in hydrogen-bonded C–H···Cl, linking two different pca^−^ ligands, which is further grown by symmetry operation, resulting in 2D hydrogen-bonded organic frameworks (HOFs) (Figure 7). If each [Co_2_(pca)_4_(bimb)] can be considered to act as a 4-connected node, then each C–H···Cl linking two [Co_2_(pca)_4_(bimb)] can be considered a linker in **2** (Figure 8). Topologically, the 2D hydrogen-bonded organic frameworks can be simplified as a 4-connected unodal ***sql*** net with a Schläfli symbol {4^4^·6^2^} (Figure 7e and Figure S2) as analyzed with the TOPOS 4.0 program [69].

**Figure 9:**
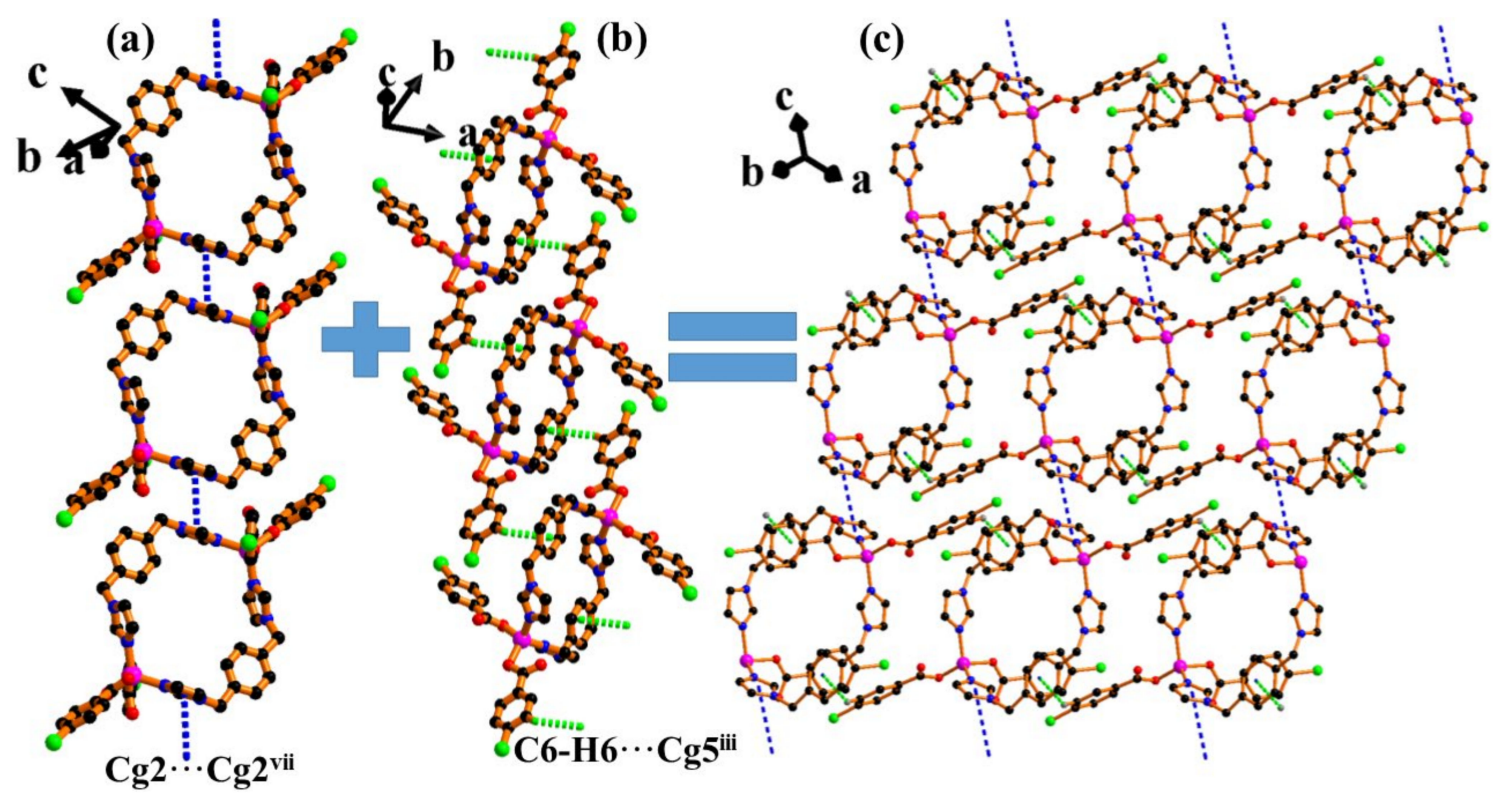
(**a**) The 1D chain constructed by π···π interactions in **2** (blue dash lines represent π···π interactions). (**b**) The 1D chain constructed by C–H···π interactions in **2** (bright green dash lines represent C–H···π interactions). (**c**) The 2D framework assembled with π···π and C–H···π interactions in **2** (blue dash lines represent π···π interactions, and bright green dash lines represent C–H···π interactions).

Furthermore, there exist two types of π-stack interactions, i.e., π···π and C–H···π, between adjacent [Co_2_(pca)_4_(bimb)_2_] units, which are non-negligible forces for stabilizing hydrogen-bonded organic frameworks. There is a π···π stacking interaction between two symmetry-related bimb ligands of imidazole rings (N3-C26-N4-C28-C27, Cg2 and Cg2^vii^; symmetry code: vii 1−*x*, −1−*y*, 2−*z*) with a Cg2···Cg2^vii^ centroid distance of 3.950(4) Å and with a dihedral angle of 0.0(4)°. These π–π stacking interactions, assembled with the binuclear structure unit into a 1D chain, approximate along the crystallographic *c*-axis (Figure 9a and Figure S3a). On a similar line, another 1D π-stacked framework along the *a*-axis direction based on the pca^−^ ligand of C–H (C6–H6) and bimb ligand of phenyl ring (C19–C20–C21–C22–C23–C24, Cg5^iii^; symmetry code: iii −*x*, 1−*y*, 1−*z*) was prepared with a pair of inversion C–H···π interactions (Figure 9b and Figure S3b). The angle of C6-H6···Cg5^iii^ is 135(3)°, and the H6···Cg2^ix^ distance is 2.95 (4) Å. These results form a 2D π-stack supramolecular framework (Figure 9c), which is further stabilized with an intermolecular non-classical hydrogen bond C–H···O. Thus, these hydrogen bonds and π-stacks insert and inter-lock the [Co_2_(pca)_4_(bimb)_2_] blocks in reversely alternating parallel arrangements, defining a 2D Co-based HOFs supporting the supramolecular architecture through C–H···O, C–H···Cl and C–H···π, π···π interactions. PLATON [60] analysis shows that the whole framework is composed of voids of 1.3% that represent 1397.3 Å^3^ per unit cell volume and 18.9 Å^3^ total potential solvent area volume.

### 2.3. Luminescent Properties 

#### 2.3.1. Solid State Fluorescence Properties of **1** and **2**

Luminescent coordination polymers (CPs) or metal-organic frameworks (MOFs) or hydrogen-bonded organic frameworks (HOFs) have received much attention, attributing the success to their potential applications in photocatalysis, biomedical imaging and fluorescent sensors [6,7,8,9,10,11,12,51,52,53,54,55,56,57,67]. The solid-state fluorescence properties of **1** and **2** were investigated at room temperature. Interestingly, complexes **1** and **2** have approximately the same excitation and emission peaks, except the intensity (Figure S4). The maximum sharp emission peaks center at 423 nm (*λ*_ex_ = 376 nm) with the intensity of 753 a.u. for **1**, and 231 a.u. for **2**, which may be attributed to the π*→n or π*→π transitions. In comparison, the emission intensity of **2** is much lower than **1** by 522 a.u., which may be assigned to different hydrogen-bonded organic frameworks. Therefore, the fluorescent ion recognition test was carried out in water, taking **1** as an example.

#### 2.3.2. Detection of Fe^3+^ Ion

**Figure 10:**
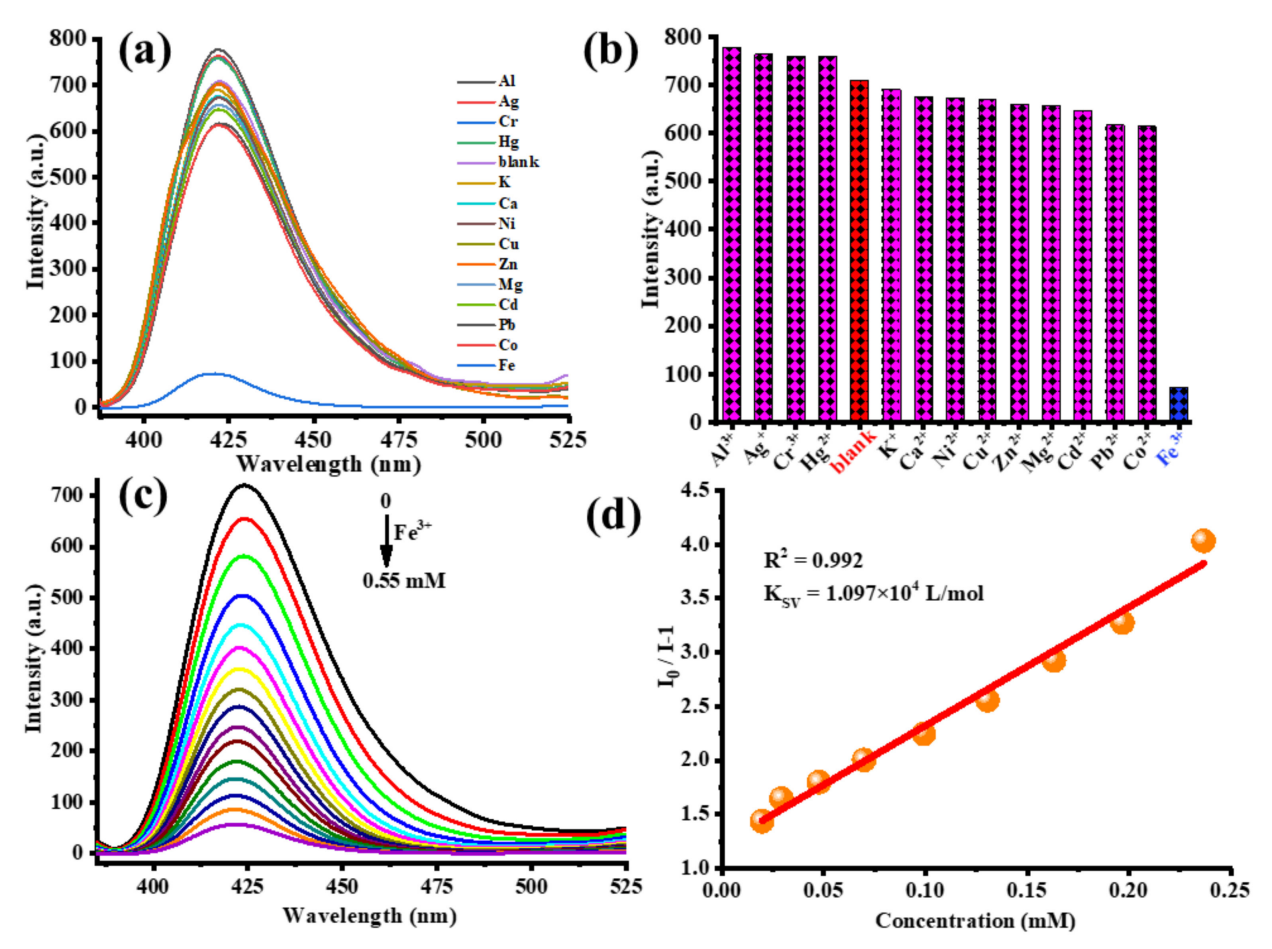
(**a**) Photoluminescence spectra of **1** in water and aqueous solutions containing different metal ions (1 × 10^−3^ M) when excited at 376 nm. (**b**) The photoluminescence intensities of **1** in aqueous solutions with various inorganic cations (1 × 10^−3^ M) when excited at 376 nm. (**c**) The emission spectra for **1** in aqueous solution of different Fe^3+^ concentrations. (**d**) The linear relationships for 1 in aqueous solution of different Fe^3+^ concentrations.

As shown in the experimental section, a variety of fluorescence spectral measurements were carried out on the fluorescent response of **1,** which interacts with 14 different metal cations. Surprisingly, the luminescence emission intensity of **1** was sharply cut down in the Fe(NO_3_)_3_ solution, but slight intensity changes were observed in the other 13 ions (Figure 10a,b). It was found that only slight enlargement or reduction changes occurred when detecting Al^3+^, Ag^+^, Cr^3+^, Hg^2+^ or Ca^2+^, Ni^2+^, Cu^2+^, Zn^2+^, Mg^2+^, while K^+^ had almost no fluorescence intensity changes. On account of the distinct response of **1** toward Fe^3+^, the quenching effect of Fe^3+^ was subsequently explored with a function of Fe(NO_3_)_3_ in the concentration range of 0–0.55 mM. As shown in Figure 10c, when the Fe^3+^ concentration was gradually raised from 0 to 0.55 mM, the fluorescence intensity from 720 a.u. decreased step by step to about 35 a.u., and the quenching efficiency was almost 95% when the concentration of the Fe^3+^ ions reached 0.55 mM. The well-known Stern-Volmer (SV) equation ((*I*_o_/*I*) = 1 + *K*_sv_[M], *I*_o_ = fluorescent intensities of **1**; *I =* fluorescent intensities after adding Fe^3+^; [M] = molar concentration of Fe^3+^ (mM); *K*_sv_ = SV constant (M^−1^)) can effectively analyze the fluorescent quenching efficiency. As shown in Figure 10d, it is clear that in the concentration range of 0.025–0.25 mM, the relative intensity has a good linear relationship with the Fe^3+^ concentration (*K*_sv_ = 10970 M^−1^, *R*^2^ = 0.992, *σ* = 0.06938). The detection limit of **1** can be calculated from the standard deviation and slope using the formula 3*σ*/*K*_sv_ (*σ*: standard error, *K*_sv_: slope). The detection limits of **1** is 19 µM. All of the above results imply that **1** can effectively and selectively sense Fe^3+^.

#### 2.3.3. Detection of Cr_2_O_7_^2−^ Anions

**Figure 11:**
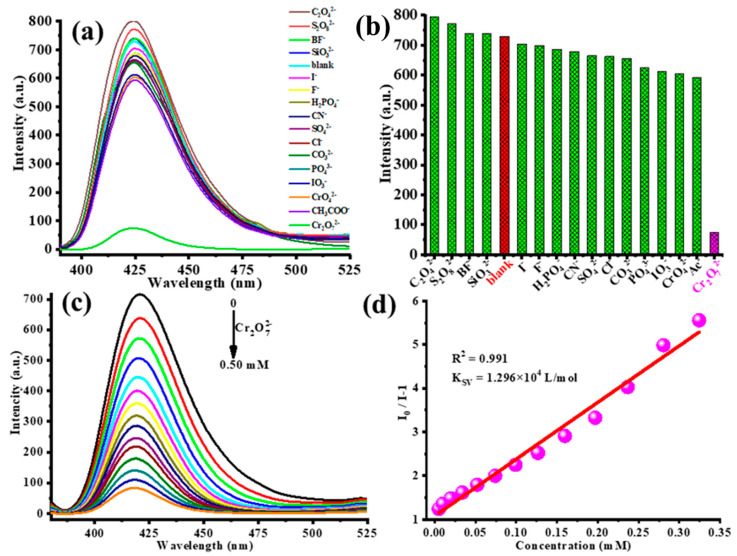
(**a**) Photoluminescence spectra of **1** in water and aqueous solutions containing different anions (1 × 10^−3^ M) when excited at 376 nm. (**b**) The photoluminescence intensities of **1** in aqueous solutions with various anions (1 × 10^−3^ M) when excited at 376 nm. (**c**) The emission spectra for **1** in aqueous solution of different Cr_2_O_7_^2−^ concentrations. (**d**) The linear relationships for **1** in aqueous solution of different Cr_2_O_7_^2−^ concentrations.

Besides metal cations, in order to improve the use efficiency of **1** as a multifunctional fluorescent probe, the fluorescence recognition anion experiments were performed to screen various potassium salt anions in aqueous solution (Figure 11a,b). Outstandingly, only Cr_2_O_7_^2-^ afforded a quite obvious luminescent quenching effect, with a quenching percentage of 93.3%, but the quenching percentage of other anions was much lower (less than 15% for Ac^−^) under the same conditions. It was also found that only slight enlargement or reduction changes occurred when detecting C_2_O_4_^2−^, S_2_O_8_^2−^ or I^−^, F^−^, H_2_PO_4_^−^, CN^−^, SO_4_^2−^, Cl^−^, CO_3_^2−^, PO_4_^3−^, IO_3_^−^, CrO_4_^2−^, Ac^−^, while BF^−^ and SiO_3_^2−^ had almost no fluorescence intensity changes. Accordingly, the titration curve clearly indicates that as the Cr_2_O_7_^2−^ concentration increases from 0 mM to 0.50 mM, and the emission intensity of **1** decreases from about 720 a.u. to 50 a.u. (Figure 11c). As shown in Figure 11d, *K*_sv_, *R*^2^ and *σ* were calculated to be 12960 M^−1^, 0.991and 0.08656 for **1**@Cr_2_O_7_^2-^, respectively. The detection limits of **1** for Cr_2_O_7_^2−^ is calculated to 20 µM based on the formulas of 3*σ*/*K*_sv_. 

#### 2.3.4. Stability and Cyclic Use Test

**Figure 12:**
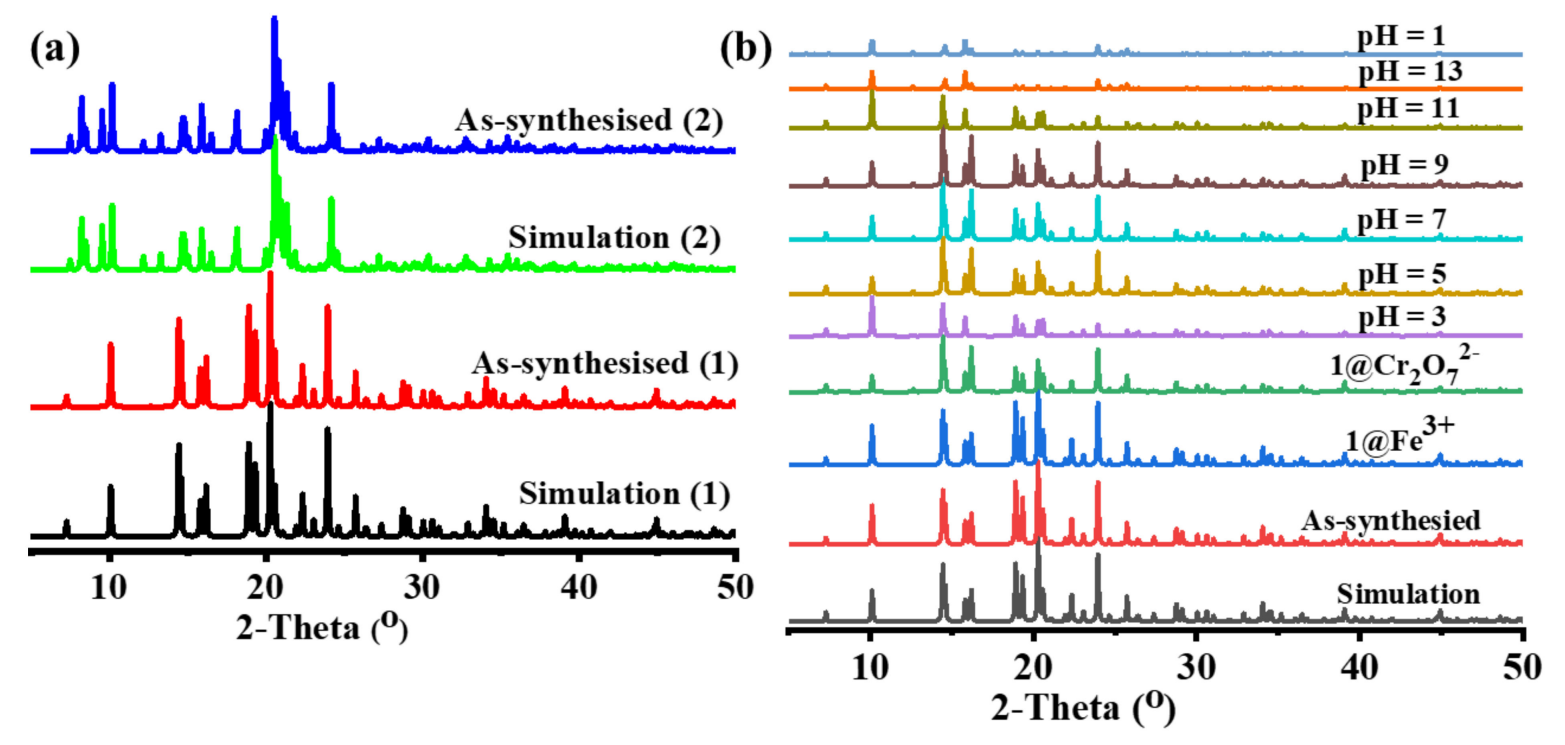
(**a**) PXRD profiles of the as-synthesized **1** and **2**, and the simulated result as reference. (**b**) PXRD profiles of the as-synthesized **1** and the simulated result as reference as well as luminescence sensing analytes, and **1** after dispersed pH = 1–13 aqueous solution.

The powder X-ray diffraction (PXRD) patterns of **1** and **2** are shown in Figure 12a. The experimental peaks are consistent with the theoretical one obtained by single-crystal X-ray data. The stability of **1** was investigated in different pH values of aqueous media varying from 1 to 13. After 24 h of soaking **1** in these solvents, the filtered samples of **1** were dried in air. The experimental PXRD patterns of **1** were also consistent with the theoretical pattern in the pH values from 3 to 11 (Figure 12b) indicating that **1** had relatively good chemical stability. The slight variation in the diffraction intensity may be related to the different crystal orientation or solvent effects. Although the experimental patterns have a few unindexed diffraction lines and some are slightly broadened in comparison to those simulated from the single-crystal model, it can still to be considered that the bulk of the synthesized materials and as-grown crystal are homogeneous, implying its excellent stability. In order to explore the thermal stability of **1** and **2**, TG studies were performed in a nitrogen atmosphere at a heating rate of 10 °C min^–1^ between 50 °C and 800 °C (Figure S5). Up to 300 °C, almost no weight loss was observed on the TG curve, which indicates the absence of an occluded solvent and guest molecules in the samples of **1** and **2**. According to TG data, heating above 350 °C leads to several different processes, which lead to the decomposition of the framework of compounds **1** and **2**.

**Figure 13:**
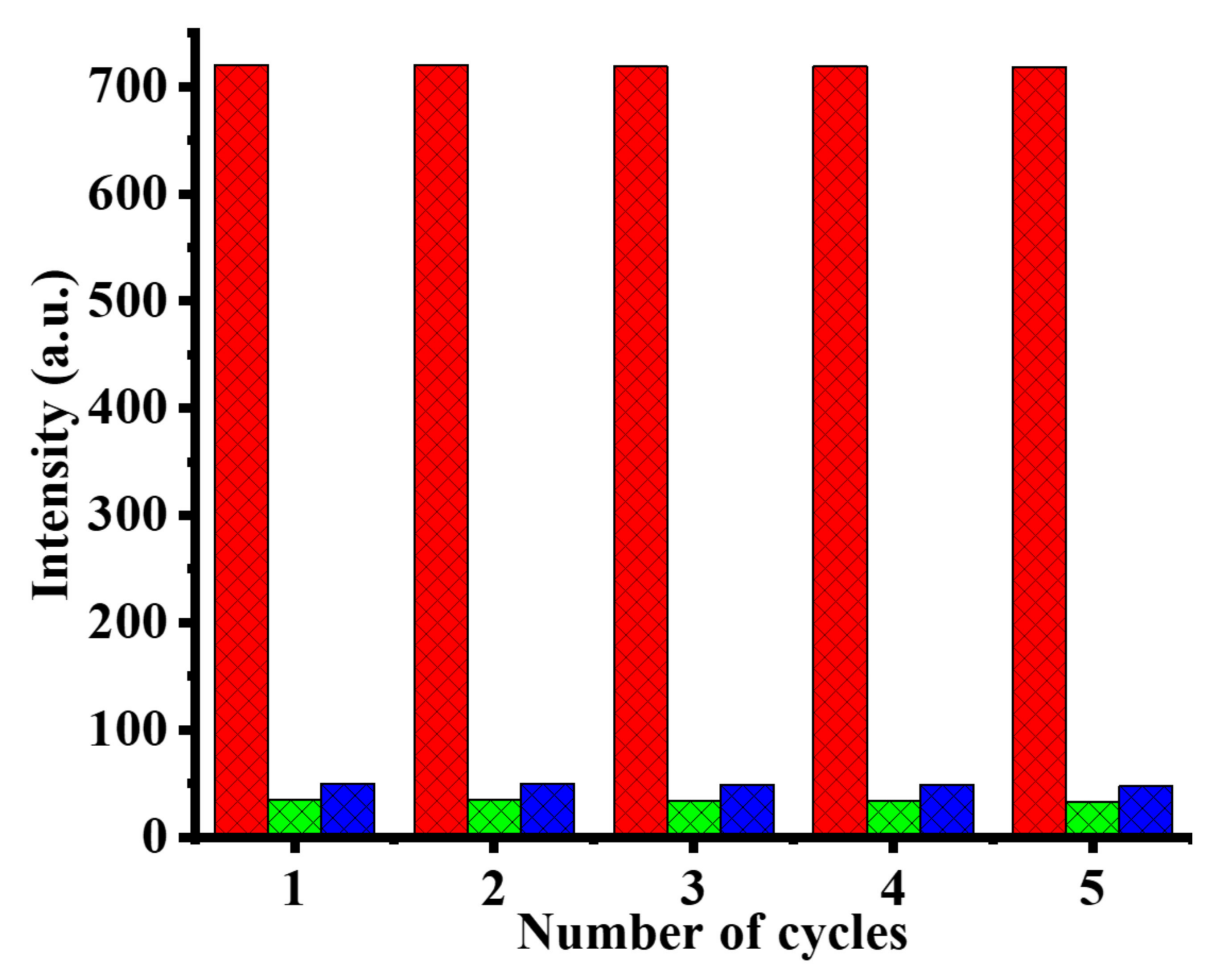
Photoluminescence intensity of **1** in five recyclable experiments for the Fe^3+^ and Cr_2_O_7_^2−^ in aqueous solution (the red bar shows the initial fluorescence intensity; the green bar shows the intensity upon addition of distilled water of 1 mM Fe^3+^ ion; and the blue bar shows the intensity upon addition of distilled water of 1mM Cr_2_O_7_^2−^ ion).

Repeatability is an important factor for assessing the applicability of luminescent coordination polymers. Recyclability experiments were conducted to determine the regeneration of **1** to detect Fe^3+^ and Cr_2_O_7_^2−^ ions. The used sample of **1** was washed multiple times with alcohol through soaking under stirring at room temperature. After filtration and drying, the regenerated sample **1** was employed for the detection of Fe^3+^ and Cr_2_O_7_^2−^ ions with the same method of **1**, respectively. It is obvious that the quenching ability of **1** for Fe^3+^ and Cr_2_O_7_^2−^ ions, respectively, are mostly unchanged for up to five cycles (Figure 13). All these results indicate that **1** has excellent water stability and reusable value.

## 3. Materials and Methods

### 3.1. Chemicals and Reagents

All chemicals and solvents, including the ligands, were purchased from Jinan Henghua Sci. & Tec. Co. Ltd. (Shandong, China) without further purification.

### 3.2. Apparatus

Powder X-ray diffraction (PXRD) data were collected on a Bruker D8 ADVANCE X-ray diffractometer (Karlsruhe, Germany) with Cu-Kα radiation (λ = 1.5418 Å) at 40 kV and 40 mA with a scanning rate of 6°/min and a step size of 0.02°. The simulation of the PXRD spectra was carried out by the single-crystal data and diffraction-crystal module of the Mercury 2.0 (Hg) program, available free of charge via the internet at http://www.iucr.org (accessed on 25 August 2021). The purity and homogeneity of the bulk products were determined by comparing the simulated and experimental X-ray powder diffraction patterns. The elemental analyses (C, H, N) were performed on a PerkinElmer 240C apparatus. The FT-IR spectra were recorded in the range of 4000–450 cm^−1^ on a PerkinElmer Frontier spectrometer (Norwalk, CT, USA). Thermogravimetric analyses (TG) were performed under nitrogen with a heating rate of 10 °C min^−1^, using a PerkinElmer Thermogravimetric Analyzer TGA4000 (Norwalk, CT, USA). Photoluminescence spectra were measured on a Varian Cary Eclipse fluorescence spectrophotometer (Shanghai, China), with a xenon arc lamp as the light source. In the measurements of emission and excitation spectra, the slit width was 10 nm. Point symbol and topological analyses were conducted by using the TOPOS program package (Samara, Russia) [69].

Single-crystal data collections were performed on a Bruker Smart Apex II CCD diffractometer (Tokyo, Japan) with graphite-monochromatized Mo*K*α radiation (*λ* = 0.71073 Å) at 296(2) K. Using Olex2 [70], the structure was solved with the ShelXT structure solution program using Intrinsic Phasing [71] and refined with the ShelXL refinement package using least squares minimization [72]. All non-hydrogen atoms were refined with anisotropic displacement parameters. Carbon-bound H atoms were placed in calculated positions (*d*_C-H_ = 0.93 Å for -CH, 0.97 for -CH_2_, and 0.96 for -CH_3_) and were included in the refinement in the riding model approximation, with *U*_iso_(H) set to 1.2*U*_eq_(C) for -CH and -CH_2_, and 1.5*U*_eq_(C) for -CH_3_. The structures were examined using the Addsym subroutine of PLATON to ensure that no additional symmetry could be applied to the models. Pertinent crystallographic data collection and refinement parameters are collated in Table S1. Selected bond lengths and angles are given in Table S2. Hydrogen-bond geometries are organized in Table S3.

CCDC 2105278 (**1**) and CCDC 2105279 (**2**) contain the supplementary crystallographic data for this paper. These data can be obtained free of charge from The Cambridge Crystallographic Data Centre via http://www.ccdc.cam.ac.uk/datarequest/cif (accessed on 25 August 2021).

### 3.3. Synthesis of [Co(pca)_2_(bmimb)]_n_ (***1***) 

A mixture of Co(CH_3_COO)_2_·4H_2_O (0.1 mmol), Hpac (0.1 mmol), NaOH (0.1 mmol), bmimb (0.1 mmol),and H_2_O–C_2_H_5_OH (5 mL/5 mL) was added to a 25 mL Teflon-lined stainless steel reactor and heated at 150 °C for 3 days, and then slowly cooled to room temperature. Red block single crystals suitable for X-ray data collection were obtained by filtration. Yield: 45% (based on cobalt acetate tetrahydrate)—Anal. for C_30_H_26_Cl_2_CoN_4_O_4_ (636.38): calcd. C 56.57, H 4.09, N 8.80; found C 56.55, H 4.08, N 8.82. Infrared (KBr pellet, cm^−1^): 3125(w), 3125(w), 1602(s), 1349(s), 1090(m), 858(m), 775(vs), 652(m), 564(m), 516(m).

### 3.4. Synthesis of [Co_2_(pca)_4_(bimb)_2_] (***2***)

The synthesis of **2** is similar to that for **1,** except for bimb (0.1 mmol) substituted for bmimb (0.1 mmol). Red block single crystals suitable for X-ray data collection were obtained by filtration. Yield: 70% (based on cobalt acetate tetrahydrate)—Anal. for C_56_H_44_Cl_4_Co_2_N_8_O_8_ (1216.65): calcd. C 55.23, H 3.62, N 9.21; found C 55.25, H 3.60, N 9.23. Infrared (KBr pellet, cm^−1^): 3644(m), 1909(w), 1711(w), 1583(s), 1500(s), 1301(m), 1083(m), 1001(m), 837 (s), 769(s), 667(m), 523(m).

### 3.5. Comparison of Synthesis and Structure for ***1*** and ***2***

Interestingly, the synthesis of **1** and **2** rely on subtle control over various hydrothermal parameters, particularly the starting neutral nitrogen-containing auxiliary ligands. This prompted us to further study this reaction system by the use of different metal ions and investigate its potential to favor the formation of higher dimensionality coordination polymers and/or MOFs. A wide range of experiments was carried out in order to study the impact of the different synthetic parameters (presence/absence or kind of base, metal ratio of the reactants, metal sources, etc.) on the identity and crystallinity of the isolated product.

The reaction mixture of Co(CH_3_COO)_2_·4H_2_O/Hpac/NaOH/bmimb (1:1:1:1) in H_2_O-C_2_H_5_OH (*v*/*v*, 1:1) at 150 °C gave a red solution from which the 1D coordination polymer [Co(pca)_2_(bmimb)]*_n_* was subsequently isolated. Following a similar reaction but using a different neutral nitrogen-containing auxiliary ligand source bimb instead of bmimb, the crystals of [Co_2_(pca)_4_(bimb)_2_] (0D) were isolated in good yield. Note that these kinds of cobalt salts do not have any impact on the identity of the isolated compound, but they affect its crystallinity. As a next step, we decided to investigate the impact of the metal ion on the identity of the isolated product by the use of Cu^2+^/Zn^2+^/Ni^2+^/Cd^2+^ and so on and following a similar synthetic approach to the one that provided access to **1** and **2**. No matter the changes and combinations used as various conditions, unfortunately, we could not obtain the target product. In comparison, we had also tried to apply the similar neutral nitrogen-containing auxiliary ligands to react with the combination of Hpca and cobalt salts under various conditions. The same results were obtained as above. 

As discussed above, two Co-based HOFs with the homologous organic ligands synthesized under the same reaction conditions show distinct structures and hydrogen bond topologies in their crystal. Using the same metal and organic carboxylic acid ligand, and different neutral auxiliary ligands but with the same synthesis strategy, will likely afford diverse structures. The biggest similarity of **1** and **2** is that they both have intermolecular non-classical hydrogen bonds C–H···Cl, which play an important role in assembling the 0 or 1D structure into the 3D supramolecular framework. Of course, the difference is also obvious between **1** and **2**. First, **1** and **2** belong to different crystal systems and space groups. Second, the steric hindrance, rigidity and electronic properties of the bmimb and bimb ligands influence the dimensionality of the reaction product. The steric hindrance and rigidity result in bigger bmimb than bimb, but the electronic properties results in better bmimb and bimb. These factors ultimately cause **1** and **2** to be formed as 1D and 0D structures, respectively, and have different hydrogen-bond framework topologies (3D for **1** and 2D for **2**).

### 3.6. Photoluminescent Sensing Experiments

The luminescence properties of **1** were investigated in the aqueous solution of various analytes at room temperature [73,74]. When measuring the emission spectra, the emission and excitation slit widths were set as 10 nm (similarly hereinafter). For the sensing of cations, 2.0 mg of a ground powder sample of **1** was immersed in 2.0 mL of a 14-cation aqueous solution of M(NO_3_)_x_ (M^x+^ = Al^3+^, Ag^+^, Cr^3+^, Hg^2+^, K^+^, Ca^2+^, Ni^2+^, Cu^2+^, Zn^2+^, Mg^2+^, Cd^2+^, Pb^2+^, Co^2+^, Zn^2+^, and Fe^3+^, 0.0010 mol·L^−1^). Then, the solid-liquid mixture was ultrasonicated for 30 min to form steady turbid suspension of **1**@M^x+^ for the fluorescence measurements. The fluorescent intensities of these **1**@M^x+^ suspensions were immediately recorded at room temperature and compared. For the sensing of 16 anions, the same procedure was used for the **1**@A^y-^ fluorescence measurements (K_y_A, A^y−^ = C_2_O_4_^2−^, S_2_O_8_^2−^, BF^−^, SiO_3_^2−^, I^−^, F^−^, H_2_PO_4_^−^, CN^−^, SO_4_^2−^, Cl^−^, CO_3_^2−^, PO_4_^3−^, IO_3_^−^, CrO_4_^2−^, Ac^−^, and Cr_2_O_7_^2−^, 0.0010 mol·L^−1^). The fluorescence intensity of the suspension **1** in water was measured as the blank sample.

### 3.7. Fluorescence Titration Experiments for Fe^3+^ and Cr_2_O_7_^2−^

The 2.0 mg ground powder sample of **1** was dispersed in 2.0 mL H_2_O solution of target analyte Fe(NO_3_)_3_ with different concentrations (0–0.55 mM). Then, the solid–liquid mixture was ultrasonicated for 30 min to form a steady turbid suspension of **1**@Fe^3+^ for the fluorescence measurements. The fluorescent intensities of these **1**@Fe^3+^ suspensions were immediately recorded at room temperature and compared. The same method was applied to measure the fluorescent spectrum of **1**@Cr_2_O_7_^2−^, with **1** soaking in different aqueous concentrations of K_2_Cr_2_O_7_ (0–0.50 mM).

### 3.8. Recyclable Luminescence Experiments

The powder of complex **1** was centrifuged from the suspension and rinsed with distilled water three times. Then, the recovered sample was dried and added to the target analytes again to measure their emission spectra. The same procedure was repeated five times.

## 4. Conclusions

In summary, two new Co-based hydrogen-bonded organic frameworks derived from the pca^−^ and bmimb/bimb ligands were hydrothermally synthesized and characterized by IR spectroscopy, elemental analysis and single-crystal X-ray diffraction. The topological structure analysis revealed that **1** and **2** are 3D or 2D Co-based HOFs with a 4-connected ***qtz*** topology with a Schläfli symbol {4^4^·6^2^} or a 4-connected ***sql*** topology with a Schläfli symbol {4^4^·6^2^} net. The luminescence investigation indicated that **1** has good fluorescence properties at room temperature and the detection limits of Fe^3+^ and Cr_2_O_7_^2−^ can reach 19 µM and 20 µM, respectively. In summary, these existing results may provide a new platform for designing luminous HOFs with multi-functional applications for detecting Fe^3+^/Cr_2_O_7_^2−^ in the future.

## Data Availability

The data presented in this study are available in supplementary material.

## Supplementary Materials

The following are available online, Figure S1: IR spectra of compounds **1** (a) and **2** (b), Figure S2: The simplified 2D HOFs for **2**, Figure S3: (a) The 1D chain constructed by π···π interactions in **2**. (b) The 1D chain constructed by C–H···π interactions in **2**, Figure S4: The solid state excitation and emission spectra of **1** and **2** at room temperature, Figure S5: TG curves of compounds **1** and **2**, Table S1: Crystal structure data for **1** and **2**^a,b,c^, Table S2: Selected bond lengths/Å and bond angles/º for compounds **1** and **2**, Table S3. Selected bond lengths (Å) and angles (deg) for **1** and **2** with estimated standard deviations in parentheses.

## Sample Availability

Samples of the compounds are not available from the authors.
