# Supplementary material for: Construction of Two Stable Co(II)-Based Hydrogen-Bonded Organic Frameworks as a Luminescent Probe for Recognition of Fe3+ and Cr2O72− in H2O
Source: Molecules. 2021 Sep 30;26(19):5955. doi: 10.3390/molecules26195955 (PMC8513017; doi:10.3390/molecules26195955)

checkCIF/PLATON report

Structure factors have been supplied for datablock(s) I

THIS REPORT IS FOR GUIDANCE ONLY. IF USED AS PART OF A REVIEW PROCEDURE FOR PUBLICATION, IT SHOULD NOT REPLACE THE EXPERTISE OF AN EXPERIENCED CRYSTALLOGRAPHIC REFEREE.

No syntax errors found.   CIF dictionary   Interpreting this report

Datablock: I

```
Bond precision:  C-C = 0.0095 A          Wavelength=0.71073

Cell:         a=10.0331(8)       b=12.8050(11)    c=13.1684(14)
              alpha=63.495(10)   beta=80.368(8)   gamma=67.362(8)
Temperature:  296 K

                Calculated                  Reported
Volume          1397.3(3)                   1397.3(3)
Space group     P -1                        P -1
Hall group      -P 1                        -P 1
Moiety formula  C56 H44 Cl4 Co2 N8 O8       C56 H44 Cl4 Co2 N8 O8
Sum formula     C56 H44 Cl4 Co2 N8 O8       C56 H44 Cl4 Co2 N8 O8
Mr              1216.65                     1216.65
Dx,g cm-3       1.446                       1.446
Z               1                           1
Mu (mm-1)       0.846                       0.846
F000            622.0                       622.0
F000'           623.50
h,k,lmax        11,15,15                    11,15,15
Nref            4973                        4961
Tmin,Tmax       0.859,0.919                 0.983,1.000
Tmin'           0.844

Correction method= # Reported T Limits: Tmin=0.983 Tmax=1.000
AbsCorr = MULTI-SCAN

Data completeness= 0.998          Theta(max)= 25.096

R(reflections)= 0.0690( 2701)     wR2(reflections)= 0.1671( 4961)

S = 1.047                  Npar= 352
```

The following ALERTS were generated. Each ALERT has the format
test-name_ALERT_alert-type_alert-level.
Click on the hyperlinks for more details of the test.

Alert level C

```
PLAT241_ALERT_2_C High   'MainMol' Ueq as Compared to Neighbors of         C6 Check
PLAT241_ALERT_2_C High   'MainMol' Ueq as Compared to Neighbors of        C13 Check
PLAT242_ALERT_2_C Low    'MainMol' Ueq as Compared to Neighbors of         C5 Check
PLAT242_ALERT_2_C Low    'MainMol' Ueq as Compared to Neighbors of         C9 Check
PLAT242_ALERT_2_C Low    'MainMol' Ueq as Compared to Neighbors of        C12 Check
PLAT334_ALERT_2_C Small Aver. Benzene C-C Dist C2       -C7              1.37 Ang.
PLAT334_ALERT_2_C Small Aver. Benzene C-C Dist C9       -C14             1.37 Ang.
PLAT334_ALERT_2_C Small Aver. Benzene C-C Dist C19      -C24             1.37 Ang.
PLAT341_ALERT_3_C Low Bond Precision on  C-C Bonds ...............     0.0095 Ang.
PLAT480_ALERT_4_C Long H...A H-Bond Reported H23      ..O1               2.91 Ang.
PLAT480_ALERT_4_C Long H...A H-Bond Reported H25A     ..O4               2.65 Ang.
PLAT480_ALERT_4_C Long H...A H-Bond Reported H27      ..O4               2.79 Ang.
PLAT481_ALERT_4_C Long D...A H-Bond Reported C23      ..O1               3.80 Ang.
PLAT910_ALERT_3_C Missing # of FCF Reflection(s) Below Theta(Min).         10 Note
PLAT911_ALERT_3_C Missing FCF Refl Between Thmin & STh/L=    0.597          2 Report
PLAT978_ALERT_2_C Number C-C Bonds with Positive Residual Density.          0 Info
```

Alert level G

```
PLAT933_ALERT_2_G Number of OMIT Records in Embedded .res File ...          2 Note
```

   0 **ALERT level A** = Most likely a serious problem - resolve or explain
   0 **ALERT level B** = A potentially serious problem, consider carefully
  16 **ALERT level C** = Check. Ensure it is not caused by an omission or oversight
   1 **ALERT level G** = General information/check it is not something unexpected

```
   0 ALERT type 1 CIF construction/syntax error, inconsistent or missing data
  10 ALERT type 2 Indicator that the structure model may be wrong or deficient
   3 ALERT type 3 Indicator that the structure quality may be low
   4 ALERT type 4 Improvement, methodology, query or suggestion
   0 ALERT type 5 Informative message, check
```

checkCIF publication errors

Alert level A

```
PUBL004_ALERT_1_A  The contact author's name and address are missing,
            _publ_contact_author_name and _publ_contact_author_address.
PUBL005_ALERT_1_A  _publ_contact_author_email, _publ_contact_author_fax and
            _publ_contact_author_phone are all missing.
            At least one of these should be present.
PUBL006_ALERT_1_A  _publ_requested_journal is missing
            e.g. 'Acta Crystallographica Section C'
PUBL008_ALERT_1_A  _publ_section_title is missing. Title of paper.
PUBL009_ALERT_1_A  _publ_author_name is missing. List of author(s) name(s).
PUBL010_ALERT_1_A  _publ_author_address is missing. Author(s) address(es).
PUBL012_ALERT_1_A  _publ_section_abstract is missing.
            Abstract of paper in English.
```

   7 **ALERT level A** = Data missing that is essential or data in wrong format
   0 **ALERT level G** = General alerts. Data that may be required is missing

Publication of your CIF

You should attempt to resolve as many as possible of the alerts in all categories. Often the minor alerts point to easily fixed oversights, errors and omissions in your CIF or refinement strategy, so attention to these fine details can be worthwhile. In order to resolve some of the more serious problems it may be necessary to carry out additional measurements or structure refinements. However, the nature of your study may justify the reported deviations from journal submission requirements and the more serious of these should be commented upon in the discussion or experimental section of a paper or in the "special_details" fields of the CIF. *checkCIF* was carefully designed to identify outliers and unusual parameters, but every test has its limitations and alerts that are not important in a particular case may appear. Conversely, the absence of alerts does not guarantee there are no aspects of the results needing attention. It is up to the individual to critically assess their own results and, if necessary, seek expert advice.

If level A alerts remain, which you believe to be justified deviations, and you intend to submit this CIF for publication in a journal, you should additionally insert an explanation in your CIF using the Validation Reply Form (VRF) below. This will allow your explanation to be considered as part of the review process.

Validation response form

Please find below a validation response form (VRF) that can be filled in and pasted into your CIF.

```
# start Validation Reply Form
_vrf_PUBL004_GLOBAL
;
PROBLEM: The contact author’s name and address are missing,
RESPONSE: ...
;
_vrf_PUBL005_GLOBAL
;
PROBLEM: _publ_contact_author_email, _publ_contact_author_fax and
RESPONSE: ...
;
_vrf_PUBL006_GLOBAL
;
PROBLEM: _publ_requested_journal is missing
RESPONSE: ...
;
_vrf_PUBL008_GLOBAL
;
PROBLEM: _publ_section_title is missing. Title of paper.
RESPONSE: ...
;
_vrf_PUBL009_GLOBAL
;
PROBLEM: _publ_author_name is missing. List of author(s) name(s).
RESPONSE: ...
;
_vrf_PUBL010_GLOBAL
;
PROBLEM: _publ_author_address is missing. Author(s) address(es).
RESPONSE: ...
;
_vrf_PUBL012_GLOBAL
;
```

```
PROBLEM: _publ_section_abstract is missing.
RESPONSE: ...
;
# end Validation Reply Form
```

If you wish to submit your CIF for publication in Acta Crystallographica Section C or E, you should upload your CIF via the web. If you wish to submit your CIF for publication in IUCrData you should upload your CIF via the web. If your CIF is to form part of a submission to another IUCr journal, you will be asked, either during electronic submission or by the Co-editor handling your paper, to upload your CIF via our web site.

PLATON version of 30/01/2018; check.def file version of 30/01/2018

Datablock I - ellipsoid plot

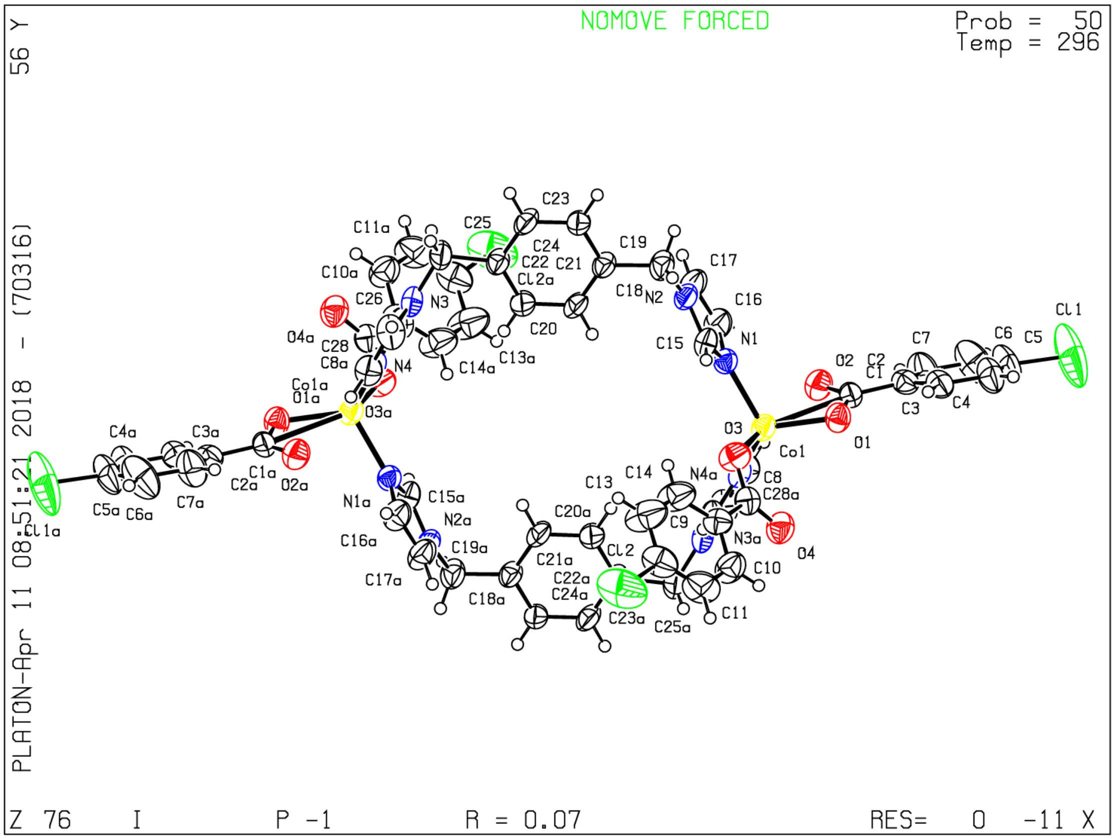

Supplement: Supplementary file 1 [file molecules-26-05955-s001.zip › 2-checkcif.pdf]
