# Supplementary material for: Construction of Two Stable Co(II)-Based Hydrogen-Bonded Organic Frameworks as a Luminescent Probe for Recognition of Fe3+ and Cr2O72− in H2O
Source: Molecules. 2021 Sep 30;26(19):5955. doi: 10.3390/molecules26195955 (PMC8513017; doi:10.3390/molecules26195955)
Supplement: Supplementary file 1 [file molecules-26-05955-s001.zip › molecules-supplementary.pdf]

Supplementary Material

Construction of Two Stable Co(II)-Based Hydrogen-Bonded Organic Frameworks as a Luminescent Probe for Recognition of Fe^{3+} and $Cr_2O_7^{2-}$ in H_2O

Qi-Ying Weng [1], Ya-Li Zhao [1,2], Jia-Ming Li [1,*], Miao Ouyang [3,*]

[1] Guangxi Key Laboratory of Green Chemical New Materials and Safety Technology, College of Petroleum and Chemical Engineering, Beibu Gulf University, Qinzhou 535011, China; qy13977735754@163.com (Q.-Y.W.); jmli@bbgu.edu.cn (J.-M.L.)

[2] College of International Studies, Beibu Gulf University, Qinzhou 535011, China; ylzhao@bbgu.edu.cn (Y.-L.Z)

[3] School of Chemical and Environmental Engineering, Hanshan Normal University, Chaozhou 521041, China; 442976264@qq.com (M.O)

* Correspondence: jmli@bbgu.edu.cn (J.-M.L); 2557@hstc.edu.cn (M.O)

Contents

*Figure S1. IR spectra of compounds **1** (a) and **2** (b).. ..…………………. 2*

*Figure S2. The simplified 2D HOFs for **2**.. ……………………….........................……..2*

*Figure S3. (a) The 1D chain constructed by π···π interactions in **2**. (b) The 1D chain constructed by C-H···π interactions in **2**.. ………………………………..…3*

*Figure S4. The solid state excitation and emission spectra of **1** and **2** at room temperature……...3*

*Figure S5. TG curves of compounds **1** and **2**. ……………………………...…..……4*

*Table S1. Crystal structure data for **1** and **2**[a,b,c]……....5*

*Table S2. Selected bond lengths/Å and bond angles/º for compounds **1** and **2**. ………….6*

*Table S3. Selected bond lengths (Å) and angles (deg) for **1** and **2** with estimated standard deviations in parentheses. ………...7*

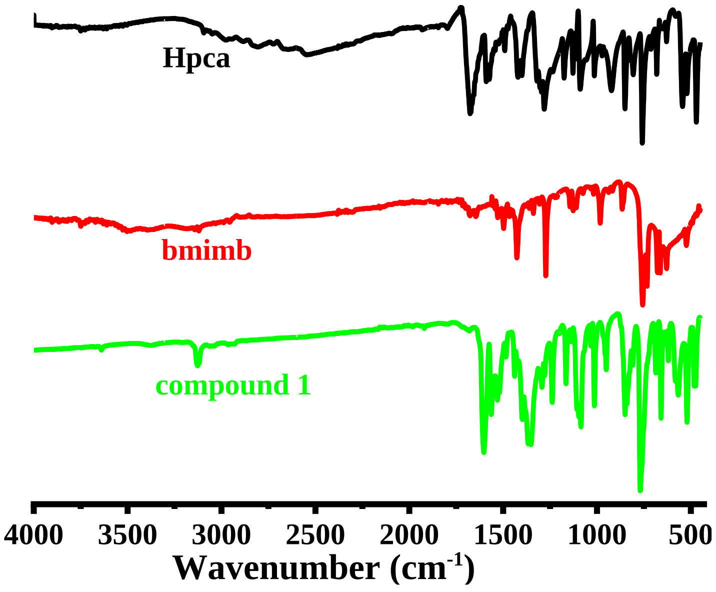

(a)

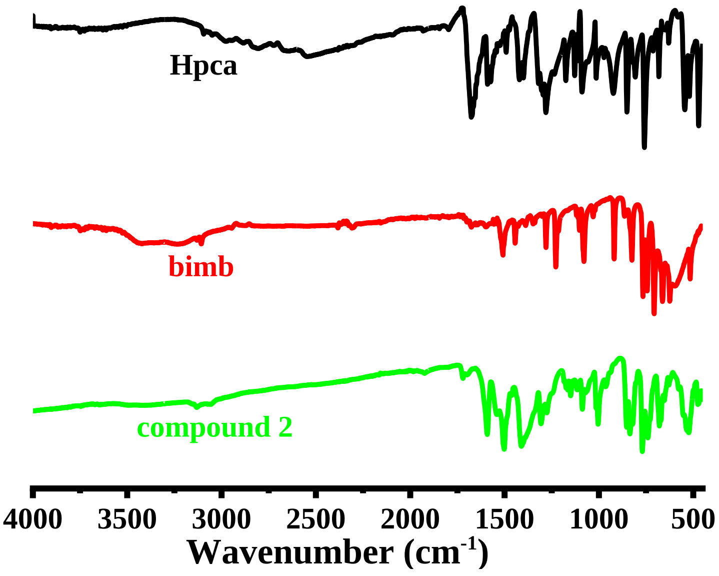

(b)

Figure S1. IR spectra of compounds **1** (a) and **2** (b).

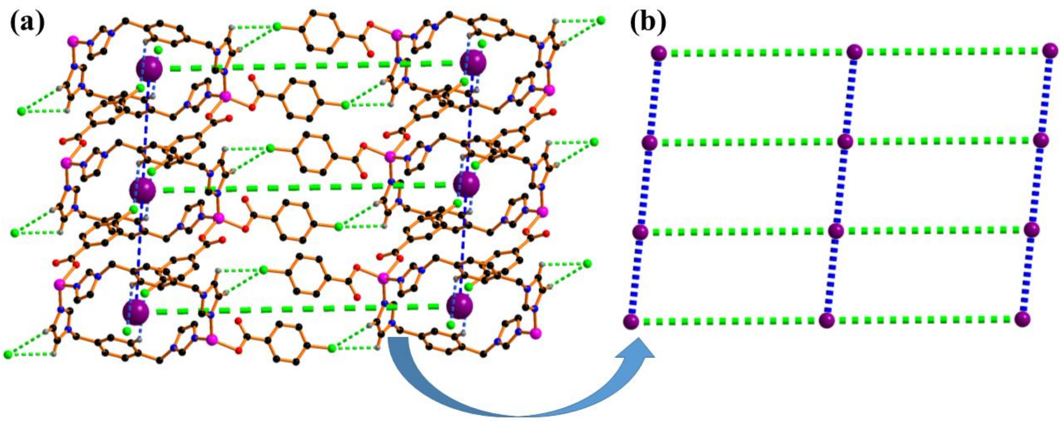

Figure S2. The simplified 2D HOFs for **2**.

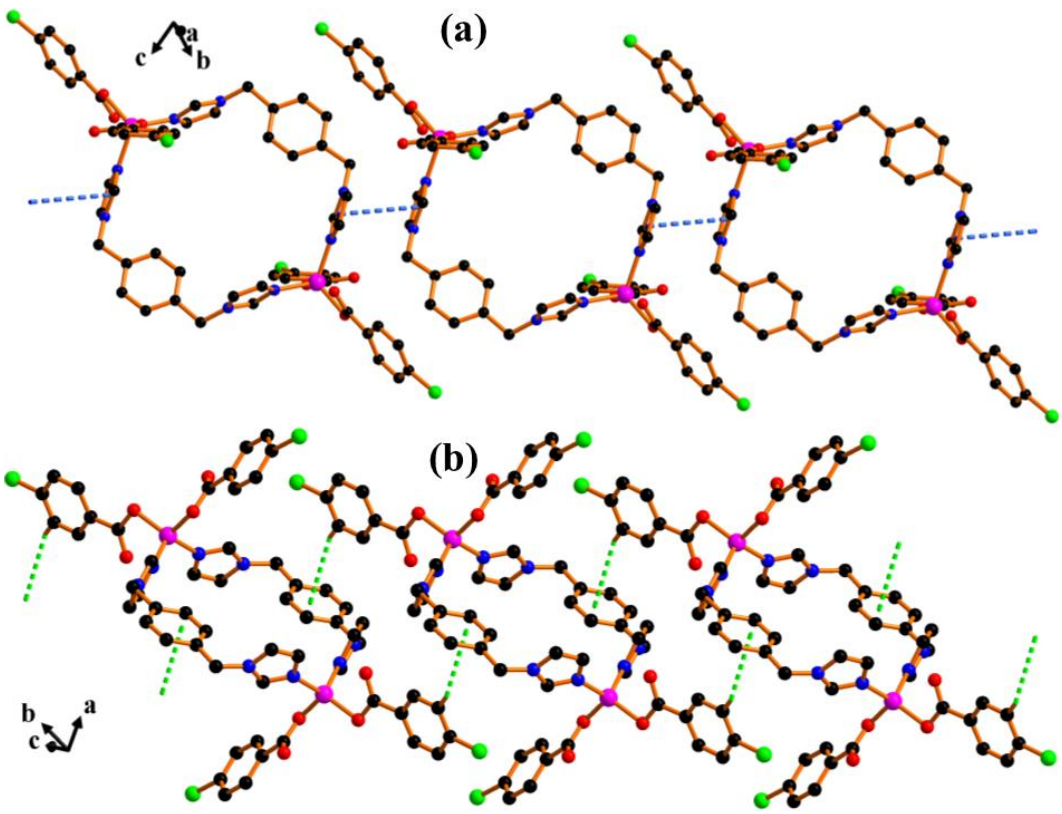

Figure S3. (a) The 1D chain constructed by π···π interactions in **2**. (b) The 1D chain constructed by C-H···π interactions in **2**.

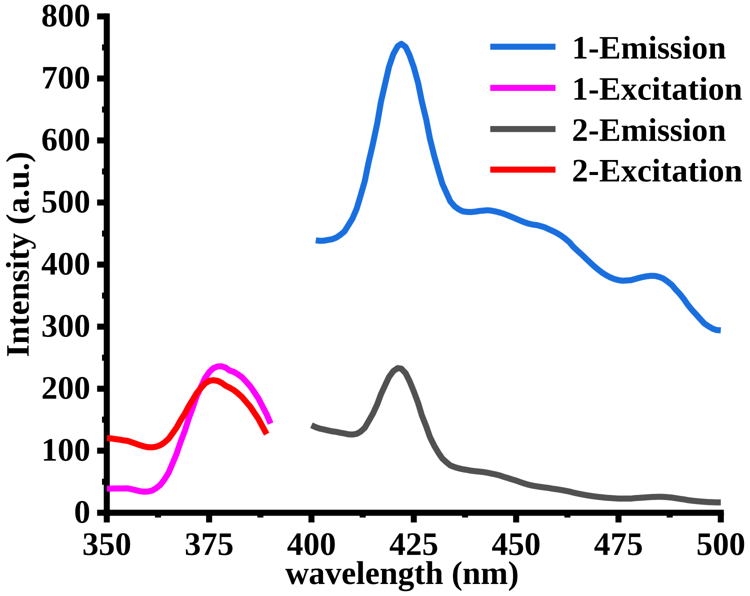

Figure S4. The solid state excitation and emission spectra of **1** and **2** at room temperature.

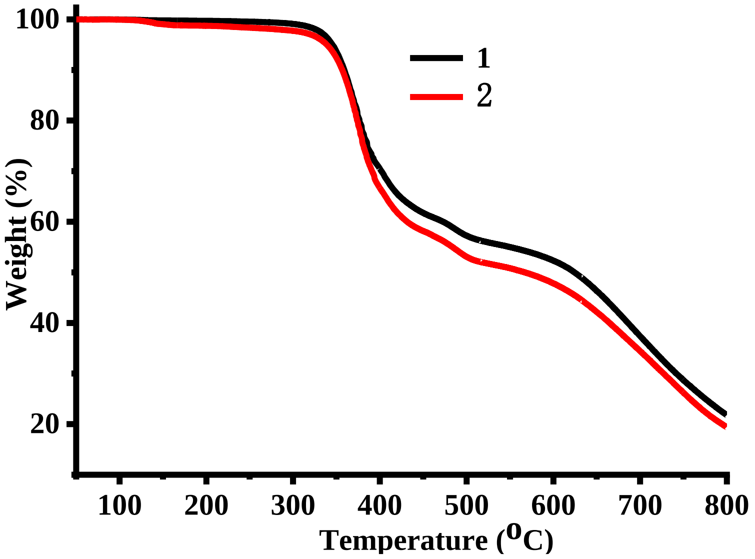

Figure S5. TG curves of compounds **1** and **2**.

Table S1. Crystal structure data for **1** and **2**[a,b,c].

Compound	**1**	**2**
Empirical formula	$C_{30}H_{26}Cl_2CoN_4O_4$	$C_{56}H_{44}Cl_4Co_2N_8O_8$
Formula weight	636.38	1216.65
Crystal system	hexagonal	triclinic
Space group	$P6_4$	P-1
a, Å	14.0240(6)	10.0331(8)
b, Å	14.0240(6)	12.8050(11)
c, Å	12.6288(6)	13.1684(14)
α, deg	90	63.495(10)
β, deg	90	80.368(8)
γ, deg	120	67.362(8)
V, Å^3	2151.0(2)	1397.3(3)
Z	3	1
D_{calcd}, g cm^{-3}	1.474	1.446
μ(MoK_α), mm^{-1}	0.828	0.846
F(000), e	981	622
hkl range	-17→16, ±17, ±15	±11, ±15, -13→15
$((\sin\theta)/\lambda)_{max}$, Å^{-1}	0.617	0.597
Refl. measured	15400	8706
Refl. unique	2827	4961
R_{int}	0.0525	0.0503
Param. refined	188	352
R_1[a] / wR_2[b] [$I > 2\sigma(I)$]	0.0400/0.0697	0.0690/0.1252
R_1[a] / wR_2[b] (all data)	0.0606/0.0799	0.1366/0.1671
GoF (F^2)[c]	1.057	1.047
$\Delta\rho_{fin}$ (max/min), e Å^{-3}	0.25/-0.34	0.54/-0.59

[a] $R = \Sigma|| F_o| - |F_c|| /\Sigma|F_o|$; [b] $wR = [\Sigma w(F_o^2 - F_c^2)^2/\Sigma w(F_o^2)^2]^{1/2}$, $w = [\sigma^2(F_o)^2 + (AP)^2 + BP]^{-1}$, where $P = (\mathrm{Max}(F_o^2,0)+2F_c^2)/3$; [c] GoF = $[\Sigma w(F_o^2 - F_c^2)^2/(n_{obs} - n_{param})]^{1/2}$.

Table S2. Selected bond lengths/Å and bond angles/º for compounds **1** and **2**.

Compound **1**[a]			
Co1-N1	2.048 (4)	Co1-N1^{i}	2.048 (4)
Co1-O1	1.948 (4)	Co1-O1^{i}	1.948 (4)
N1-Co1-N1^{i}	111.5 (2)	O1-Co1-N1	117.81 (16)
O1-Co1-N1^{i}	92.58 (15)	O1i-Co1-N1^{i}	117.81 (16)
O1^{i}-Co1-N1	92.58 (15)	O1-Co1-O1^{i}	126.0 (2)
Compound **2**[b]			
Co1-O1	1.978 (3)	Co1-O3	1.961 (3)
Co1-N1	2.013 (4)	Co1-N4^{i}	2.028 (5)
O1-Co1-N1	111.84 (16)	O1-Co1-N4^{i}	116.81 (16)
O3-Co1-O1	110.89 (15)	O3-Co1-N1	95.96 (16)
O3-Co1-N4^{i}	105.53 (17)	N1-Co1-N4^{i}	113.56 (17)

[a]Symmetry codes: i −*x*+2, −*y*+1, *z*; ii −*x*+1, −*y*+1, *z*.

[b]Symmetry codes: i −*x*+1, −*y*, −*z*+1.

Table S3. Selected bond lengths (Å) and angles (deg) for **1** and **2** with estimated standard deviations in parentheses.

D-H···A	d(D-H)	d(H···A)	d(D...A)	∠(DHA)
Compound **1**[a]				
C16-H16···O2^{iii}	0.93	2.95	3.728 (8)	142
C16-H16···O2^{iv}	0.93	2.95	3.728 (8)	142
C15-H15···N1^{v}	0.93	2.89	3.682 (7)	144
C15-H15···N1^{vi}	0.93	2.89	3.682 (7)	144
C4-H4···Cl1^{vii}	0.93	2.95	3.743 (6)	144
C8-H8A···Cl1^{viii}	0.96	2.84	3.676 (7)	147
Compound **2**[b]				
C15-H15···O1^{ii}	0.93	2.47	3.261 (6)	142
C17-H17···O2^{iii}	0.93	2.51	3.417 (7)	165
C18-H18B···O1^{ii}	0.97	2.60	3.491 (7)	152
C23-H23···O1^{iv}	0.93	2.91	3.798 (6)	161
C25-H25A···O4^{iv}	0.97	2.65	3.539 (7)	153
C27-H27···O4^{iv}	0.93	2.79	3.425 (8)	126
C27-H27···Cl1^{v}	0.93	3.175	3.522 (6)	104
C28-H28···Cl1^{v}	0.93	3.125	3.511 (6)	107
C20-H20···Cl2^{vi}	0.93	3.308	3.884 (6)	122

[a]Symmetry codes: iii −*y*+1, *x*−*y*, *z*−2/3; iv *y*, −*x*+*y*+1, *z*−2/3; v −*y*+1, *x*−*y*, *z*+1/3; vi *y*, −*x*+*y*+1, *z*+1/3; vii −*x*+2, −*y*+2, *z*; viii −*x*+*y*+1, −*x*+2, *z*−1/3.

[b]Symmetry codes: ii −*x*+1, −*y*+1, −*z*+1; iii −*x*, −*y*+1, −*z*+1; iv *x*, *y*−1, *z*+1; v *x*+1, *y*-2, *z*+1; vi *x*-1, *y*, *z*.
